# New insights into enhancement of cadmium biosorption from industrial wastewater through *Chlorella sorokiniana* HMYA based thin-film

**DOI:** 10.1039/d5ra06679d

**Published:** 2025-12-16

**Authors:** Heba M. Youssef, Fatma Mohamed, Mohamed S. Abd Elhameed, Khaled N. M. Elsayed

**Affiliations:** a Botany and Microbiology Department, Faculty of Science, Beni-Suef University 62511 Egypt k.elsayed@science.bsu.edu.eg; b Nano Photonics and ApplicatVions Lab, Faculty of Science, Beni-Suef University Beni-Suef 62514 Egypt; c Materials Science Research Laboratory, Chemistry Department, Faculty of Science, Beni-Suef University Beni-Suef Egypt; d Biology Department, Faculty of Education and Arts, Sohar University Sohar 311 Oman kelsayed@su.edu.om

## Abstract

Cadmium (Cd^2+^) is a non-essential and highly toxic heavy metal released from industrial and agricultural activities such as electroplating, dyeing, battery manufacturing, fertilizer application, fuel combustion, and cigarette smoke. Its environmental persistence leads to bioaccumulation and food chain transfer, posing severe teratogenic, carcinogenic, and neurotoxic risks to ecosystems and human health, necessitating the development of sustainable remediation strategies. We present a novel biosorption system for industrial wastewater using *Chlorella sorokiniana HMYA-C* based thin film, demonstrating high biosorption efficiency with strong potential for large-scale applications. This novel thin film outperformed both wet biomass and traditional immobilized beads under optimal conditions (pH 7, 25 °C, 2.3 g biomass/16 ml alginate/50 ml metal solution). The algal thin film successfully removed 100%, 80%, 70%, and 64% of Cd^2+^ from aqueous solutions at an initial concentration of 10, 20, 50, and 80 ppm. Furthermore, it remarkably eliminated all of the cadmium from real industrial wastewater which containing 0.4 and 2.4 ppm of Cd^2+^ concentration, highlighting its potential for immediate deployment as a biotechnological tool. After biosorption, the Cd-loaded *Chlorella sorokiniana HMYA-C* thin film can be safely using mild acidic or chelating agents or converted into biofuels under regulated conditions, while advancing circular bioeconomy principles. Material characterization (FTIR, EDX, SEM, XRD, zeta potential) indicated a porous, heterogeneous surface capable of multilayer adsorption compatible with pseudo-second-order kinetics and Freundlich isotherms. Overall, this innovative microalgae based thin film platform shows great promise for industrial scalability, addressing major economic and environmental concerns while meeting pressing global demands.

## Abstract 2

*Chlorella sorokiniana HMYA* thin film is an eco-friendly, cost-effective biosorbent, achieving up to 100% Cd^2+^ removal from real wastewater, offering scalable, sustainable solutions for industrial biosorption.

## Graphical abstract

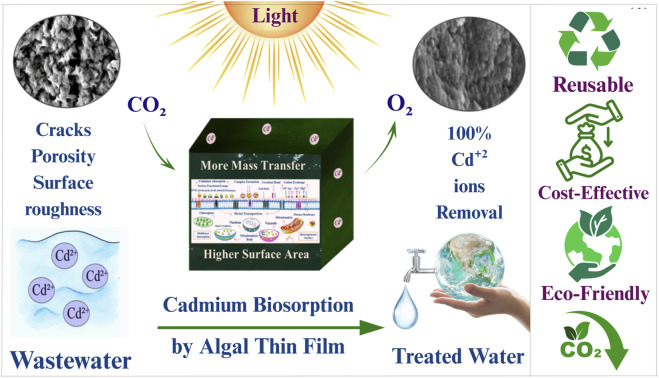

## 1 Introduction

Water is a key resource that is critical to human life and environmental stability.^1^ Rapid industrialization, population growth, and severe implications of climate change resulted in an extreme water issues.^2^ According to United Nations forecasts, by 2025, approximately 1.8 billion people will be living in regions with absolute water scarcity.^3^ Continuous discharge of untreated or inadequately treated industrial wastewater into natural water systems exacerbates the issue.^4^ Among the diverse contaminants in wastewater, heavy metals are particularly concerning due to their severe toxicity, environmental persistence, and resistance to degrade naturally.^5–9^ Cadmium is regarded as one of the most dangerous heavy metals due to its higher mobility, prolonged biological half-life, and substantial effects at extremely low doses.^10–14^ The World Health Organization restricts cadmium levels in drinking water to 3 µg L^−1^.^15,16^ Conventional physicochemical treatment technologies, such as chemical precipitation, ion exchange, membrane filtration, and electrochemical methods are widely employed for removing cadmium from wastewater.^17–19^ However, these technologies are typically associated with high operational expenses, significant energy consumption, lack the selectivity and complex procedures.^20–24^ These restrictions prompted researchers to look into alternative eco-friendly remediation solutions, with biological techniques appearing particularly promising.^25,26^ Among biological remediation techniques, phycoremediation, or the use of microalgae to remove toxins, is gaining appeal in biological systems because of its remarkable adaptability, photosynthetic efficiency, rapid development, and metabolic plasticity.^27–35^ The presence of functional groups (such as carboxyl, hydroxyl, sulfate, and phosphate) in microalgal cell walls promotes metal biosorption detoxification and bioaccumulation; the effectiveness of these processes varies depending on the species, environmental factors, and type of metal.^36–39^ In particular, carboxyl and hydroxyl groups were primarily involved in ion-exchange and electrostatic interactions, whereas amine and phosphate groups contributed mainly through coordination complexation with Cd^2+^ ions. Furthermore, their dual role in pollution removal and biomass valorization into high value products strengthens its scalability within circular bioeconomy frameworks.^40–43^ Despite their potential, traditional harvesting methods (such as centrifugation, filtration, and flocculation) are energy intensive and costly.^44^ Immobilization techniques offer a scalable alternative that enhances microalgal stability, recyclability, and ease of separation. This study employed sodium alginate and calcium chloride to produce immobilized beads and thin films of the selected microalgae. Table 1 compares different microalgal designs employed in heavy metal bioremediation, highlighting variations in biosorption efficiency arising from differences in the physical structure of the biosorbent. The comparison clearly indicates that the immobilized *Chlorella sorokiniana HMY-C* thin film developed in this study achieved the highest cadmium removal efficiency (100%), significantly outperforming conventional microalgal designs. This demonstrates the critical role of immobilized thin film structure in enhancing surface area, functional group accessibility, and biosorption performance.

**Table 1:** Shows the comparison between the microalgal designs utilized in heavy metal bioremediation, highlighting differences in biosorption efficacy

Microalgal strain	Technique	Heavy metal	Removal efficiency %	Ref.
*Chlorella sorokiniana* HMY-C	Immobilized thin film	Cd^2+^	100%	This study
*C. pyrenoidosa*	Wet biomass	Cd^2+^	45.45%	45
*Scenedesmus acutus*			57.14%	
*Desmodesmus* sp.	Wet biomass	Cu^2+^	95%	46
		Ni^2+^	90%	
*Chlorella* sp.	Immobilized beads	Pb^2+^	> 90%	47
*Chlorella vulgaris*	Wet biomass	Zn^2+^	99.4%	48
		Cu^2+^	91.9%	
*Chlorophyceae* spp.	Wet biomass	As	88%	
*Chlorella sorokiniana*	Immobilized beads	Cu^2+^	97.10%	49
		Cd^2+^	64.61%	
*Tetradesmus obliquus*	Immobilized beads	Cd^2+^	99.85%	50
*Dunaliella salina*	Wet biomass	Pb^2+^	87.2%	51
		Cd^2+^	72.9%	
		Cu^2+^	88.9%	
*Chlorella vulgaris*	Immobilized beads	Cd^2+^	100%	52
		Pb^2+^	100%	
*Turbinaria ornata*	Dry biomass	Cd^2+^	94.34%	53
	Immobilized beads		98.65%	
*Chlorella sorokiniana*	Dry biomass	Cu^2+^	90.7%	54
		Zn^2+^	87.1%	
*Scenedesmus* sp.	Dry biomass	Pb^2+^	85%	55
		Cd^2+^	83%	

**Fig. 1:**
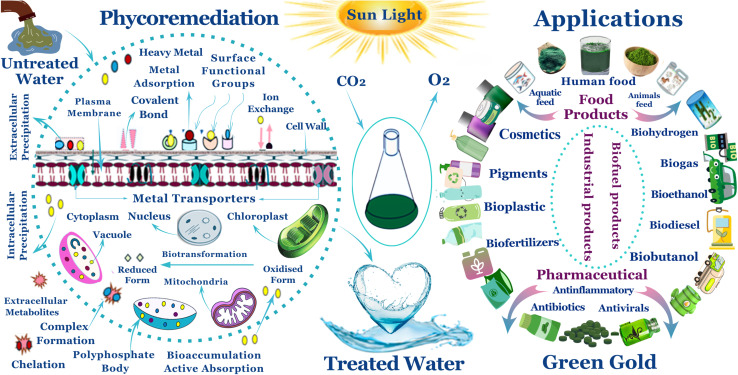
An illustration of the microalgae benefits in both environmental remediation and sustainable resource management. This emphasizes the phycoremediation process, which employs algae to remove heavy metals from untreated water. Highlighting crucial processes such extracellular precipitation, covalent bonding, ion exchange, and bioaccumulation. The treated water is safe to reuse, and the algal biomass generated may be utilized to manufacture biofuels, medications, biofertilizers, cosmetics, and other products.

Alginate, a biodegradable and non-toxic polysaccharide generated from brown seaweed, acts as a protective matrix, enhancing nutrient and light penetration, decreasing cell aggregation, and improving bio-sorption efficiency.^56–60^ Calcium chloride (CaCl_2_) is a crosslinking agent that promotes fast gelation by increasing ionic contacts between divalent Ca^2+^ ions and alginate chains' carboxylate groups, so it acts as a hardening agent for the different shapes of immobilized alginate.^61,62^ Thin films, in particular, have demonstrated more effective biosorption kinetics and metal absorption efficiency than conventional wet biomass and bead based systems, due to their higher surfaces, long-term stability, better mass transfer, and greater functional group interactions with cadmium ions. Furthermore, they shown increased hydrogen generating capabilities.^63^ This dual functionality emphasizes the potential of algal thin films as a sustainable platform that enables efficient wastewater remediation while simultaneously generating biomass for biofuels, pharmaceuticals, fertilizers, cosmetics, and other valuable products, Fig. 1.

Although *Chlorella sorokiniana HMY-C* thin films demonstrated promising cadmium removal capabilities ready-to-deploy potential, a comparison with both conventional and emerging  technologies is still necessary, as shown in Table 2. Even with their effectiveness, emerging techniques like nanomaterials and genetically modified microorganisms have drawbacks in terms of cost, scalability, and safety issues.^64–68^ Despite growing interest in algal-based remediation, the potential of microalgae thin-film biosorption systems remains unexplored. Conventional microalgal bioremediation employs large open ponds containing suspended cells, exhibit low recyclability, and pose concerns of microbial leakage into natural ecosystems. Immobilized thin-film systems, on the other hand, provide a restricted yet metabolically active environment that boosts biosorption efficiency, operational stability, and reusability. This study fills a significant gap by assessing the biosorptive capacity, scalability, and real-world feasibility of *Chlorella sorokiniana HMYA-C* thin films for sustainable cadmium removal from industrial wastewater. This study aimed to isolate and purify native green microalgae from Beni-Suef Zoo in Egypt and assess the potential of promised strain for cadmium phycoremediation. A comparative evaluation of wet biomass, immobilized beads, and algal thin film was carried out. The cadmium removal efficiency using algal thin film from real industrial wastewater which containing 0.4 and 2.4 ppm of Cd^2+^ concentration was determined. The performance and mechanisms of Cd(ii) removal from aqueous solutions were examined using batch experiments and sophisticated characterization techniques, such as SEM, EDX, FTIR and zeta potential with isotherm and kinetic modeling.

The comparison between microalgal-based thin film biosorption with conventional cadmium removal technologies (chemical precipitation membrane filtration (MF), activated carbon adsorption, ion exchange) and other emerging technologies (nanotechnology-based adsorbents or genetically engineered microorganisms), emphasizing mechanisms, performance, cost, environmental impact, and operational viability

**Table:** 

Technology	(Microalgal based thin film)	Chemical precipitation	Membrane filtration (MF)
Conceptual mechanism	Immobilized microalgae on thin films facilitate biosorption *via* evaluation of microalgal biosorptive characteristics	Chemical precipitants, such as CaO and Na_2_S, are used to transform soluble Cd^2+^ ions into insoluble compounds^69–71^	Membranes segregate contaminants *via* size exclusion or charge interaction through porous barriers^72^
Cd^2+^ removal efficiency %	Removed 100%, 80%, 70%, and 64% of Cd^2+^ for 10, 20, 50, and 80 ppm (synthetic); 100% removal at 2.4 ppm in real industrial effluent (this study)	Sulfide precipitation process removed 85.6% of Cd^2+^ (ref. 73). Calcium oxide precipitation removed 99.9% of Cd^2+^ (ref. 74)	Removal 96% of Cd^2+^*via* micellar-enhanced UF.^75^ Up to 98% elimination with reverse osmosis.^76^ 97.63% of Cd^2+^ by clam shell-based membrane
Total cost (USD m^−3^)	(∼$ 0.8–1.11 m^−3^, this study)	($ 4 m^−3^)^77^	(∼$ 0.47 m^−3^, as reported (0.44 €) in 78.) ∼ $ 0.47 m^−3^, as reported (0.44 €) in ref. 81
Scalability in practice	Still at the pilot and semi-industrial stages; extendable using modular biofilm modules	Highly scalable; often utilized in large-scale wastewater systems^79^	Widely used in industrial and municipal wastewater systems^70,80–82^
Environmental impact	Eco-friendly, reduces CO_2_ emissions. Biomass might be used for bioenergy	Creates significant amounts of chemical sludge, which must be disposed of or treated^83^	Little chemical waste; membrane disposal and cleaning chemicals offer mild environmental problems^84^
Reusability potential	Fast desorption and several reuses of the biosorbent	No reusability: continual chemical input is required^85^	Membranes may be reused after cleaning; however their performance diminishes with fouling and chemical assault^86^
Operational challenges	Thin film contamination, biofouling	Demands strict control of pH and stoichiometry; sludge processing is labor-intensive^87^	Membrane fouling, scaling, and pressure loss necessitate frequent maintenance and pre-treatment procedures^17^

**Table:** 

Activated carbon adsorption	Ion exchange	Nanotechnology	Genetically engineered microorganisms
High surface area (500–1500 m^2^ g^−1^) and porous structure allow for physical adsorption of metal ions^88^	Charged polymeric resins exchange target metal ions for benign counterions in a reversible, selective process^89^	Nanomaterials (*e.g.*, nanotubes, oxides) provide high reactivity and selective adsorption because of designed surface functions	Engineered microorganisms employ metal-binding peptides or improved efflux mechanisms to absorb, sequester, or convert hazardous metals^90^
Up to 98% of Cd^2+^ using active carbon prepared from sunflower seed shell. Up to 85% of Cd^2+^ utilizing rhus pentaphylla sulfuric acid^91^	Amberlite IR120H resins removed 96% of Cd.^92^ Ca(OH)_2_ and Mg(OH)_2_ Modified Amberlyst15 remove 99% of Cd^93^	Up to 92.5% using nanocellulose (NC).^94^ Up to 93% using chitosan-grafted poly (carboxymethyl cellulose-Co-acrylamide) nano hydrogel.^95^	Engineering bacteria demonstrated a survival rate >70% of Cd^2+^, whereas wild bacteria's survival rate remained > 50%.^96^ Up to 80% using engineered *Escherichia coli* cell factory^97^
($ 5–200 m^−3^,^98^ as cited in ref. 99)	($ 0.237 m^−3^)^100^	($ 6.35 m^−3^)^101^	Currently limited to lab-scale research; no industrial cost estimates are available
Scalable, but requires development of cost-effective synthesis methods^102^	Effective, but requires well-regulated operating settings^103^	Integrated into existing hybrid treatment systems, modular deployment feasible^104^	Large-scale optimization is required for full industrial adoption^105^
Although the usage of bio-carbon is environmentally favorable, waste creation may be an issue^106^	Resin production and regeneration. Consume water and create waste streams^26^	Engineered surfaces reduce toxicity and, when correctly managed, have a minimal ecological imprint^107^	Low environmental load; enhances biodegradability and bioremediation capability^108^
Adsorbents can be thermally or chemically regenerated several times^106^	Depends on resin type, and efficiency decreases with each cycle^109^	Nanomaterials can be regenerated; however, aggregation and leaching must be addressed^110^	Microbial systems may be regenerated through growth cycles and genetic improvements^111^
Fouling, and monitoring of the feed stream, as well as factors such as pH and temperature to ensure consistent performance^112^	Regular resin replacement; susceptible to clogging and scaling^113^	Nanoparticle aggregation, leaching, and recovery all offer engineering issues^114^	Maintaining microbial viability, mutation stability, and avoiding gene leaking^115^

## 2 Materials and methods

### 2.1 Materials

Wuxal media (universal fertilizer, Wilhelm Haug GmbH and Co. KG, Germany) and deionized water (Botany lab, Beni-Suef university, Egypt). Sodium alginate (MW = 380.000 g mol^−1^, 0.1 M, Sigma-Aldrich). Cadmium sulfate (3CdSO_4_·8H_2_O, 99%, Oxford Laboratory Reagent, INDIA). Calcium chloride (CaCl_2_, MW = 110.984 g mol^−1^, 96%, 0.18 M, Piochem). Taq buffer (Ferments, Germany). DNA gel extraction kit from (Sigma-Aldrich, Germany).

### 2.2 Purified microalgal strains preparation

#### 2.2.1 Sample collection

Soil and water samples were collected from Beni-Suef Zoo, Beni-Suef governorate, Egypt, located at latitude: 28° 53′ 37.981″ N and longitude: 31° 26′ 44.224 E, using different sampling techniques include inverted Petri dishes, scraping, brushing, and syringe sampling in sterile equipment like 50-ml tubes and Petri dishes. All collected samples were maintained under refrigerated conditions during their transport to the phycology laboratory at the Faculty of Science, Beni-Suef University, Egypt.

#### 2.2.2 Pre-isolation techniques

Collected water samples were treated with nutritional medium in a laboratory environment, to enhance microalgae growth before isolation.^116^ The microalgal biomass was concentrated by centrifugation at 3000 rpm for 5 minutes, allowing microalgal cells to be isolated from the bulk medium *via* gravity.^117^ Pre-isolation techniques were applied to enhance microalgal growth and concentrate biomass, facilitating successful isolation of strains with strong cadmium biosorption potential.

#### 2.2.3 Isolation and purification of microalgae

Pure Unialgal strains were isolated by repeated dilution and streak plate methods. A tenfold serial dilution was performed by mixing 1 ml of homogenized samples with 9 ml of sterile culture material in six sterile test tubes. Each dilution step was properly mixed, and the tubes were cultivated for 14 days at 25 °C.^118^ Aliquots of each dilution were plated onto sterile Petri plates containing 20 ml of solidified nutritional agar in an aseptic laminar flow hood using standard streaking and zigzag inoculation techniques. Frequent streaking was performed to ensure the isolation of pure strains.^119^ The isolation and purification steps ensure that only pure, viable microalgal strains are selected, which is essential for accurate assessment of biosorption capabilities.

#### 2.2.4 Assessment of isolated strains purity

To assess the purity of the isolated unialgal strains, 100 µL of each culture was placed on nutrient agar plates (NA), a typical medium for bacterial growth. Plates were incubated at 37 °C for 48 hours and examined for the presence of bacterial colonies. The purity of the algal cultures was verified by the absence of bacterial contamination. Furthermore, a microscopic examination was conducted to ensure the absence of non-algal contaminants. Confirming the absence of contaminants guarantees that biosorption analyses reflect the properties of the microalgae alone, supporting reliable results.

#### 2.2.5 Molecular identification of microalgae

##### 2.2.5.1 Genomic DNA extraction

Genomic DNA was isolated from pure unialgal cultures using the DNeasy Plant Mini Kit (Qiagen, Germany) according to the manufacturer's directions. DNA content and purity were measured spectrophotometrically at 260 and 280 nm. The extracted DNA's integrity was confirmed using electrophoresis on a 1% agarose gel stained with ethidium bromide. DNA bands were visible during UV transillumination, confirming the existence of intact genetic material.

##### 2.2.5.2 PCR amplification of the 18S gene

PCR amplification of the 18S gene was carried out with species-specific primers: DSs (5′-GCAGGAGAGCTAATAGGA-3′) and DPs (5′-GTAGAGGGTAGGAGAAGT-3′). The reaction mixture (25 µL) includes 0.2 µL Taq DNA polymerase, 1 µL genomic DNA (∼10 ng), 2.5 µL dNTPs, 2.5 µL of each primer (10 pmol µL^−1^), 5 µL of 10× Taq buffer, and nuclease-free water to the final volume.^120^ The PCR cycling conditions were as follows: 4 minutes of initial denaturation at 94 °C, 35 denaturation cycles lasting 50 seconds each, 60 seconds of annealing at 58 °C, 1 minute of extension at 72 °C, and 7 minutes of final extension. Agarose gel electrophoresis was used to resolve the amplified products, which were subsequently purified using a commercial DNA gel extraction kit for future use.

##### 2.2.5.3 DNA sequence

Purified PCR products were sequenced bidirectionally using the BigDye® Terminator v3.1 Cycle Sequencing Kit (Applied Biosystems, USA), according to the manufacturer's instructions. Macrogen Inc. (Seoul, South Korea) used an ABI Prism 310 Genetic Analyzer from Applied Biosystems to execute sequencing procedure. The raw sequence data was examined for quality, and any ambiguous base calls were manually curated using Chromas software (Technelysium Pty Ltd).

##### 2.2.5.4 Phylogenetic analysis

The obtained 18S rRNA gene sequences were compared to existing entries in the NCBI GenBank database using the Basic Local Alignment Search Tool (BLAST) to determine their closest taxonomic relatives.^121^ Multiple sequence alignments were carried out with MUSCLE, which is part of the MEGA 11 software suite. To assess branch support, phylogenetic trees were built using the Maximum Likelihood method and 1000 bootstrap replications. All analyses were carried out using MEGA11 software (Molecular Evolutionary Genetics Analysis version 11).^122^ Representative sequences were uploaded to GenBank and assigned accession numbers.

#### 2.2.6 Cultivating pure unialgal strain

Pure unialgal strains were cultivated in BG11 and Wuxal medium in a controlled laboratory environment. Cultures were maintained at a temperature of 25 ± 2 °C and pH 7.0 under continuous illumination with cool white, fluorescent light 24: 0 h light/dark cycle at an intensity of ∼60 µmol photons m^−2^ s^−1^. Microalgae were cultured in sterile 250-ml Erlenmeyer flasks with 100 ml of media, aerated with sterile air pumps for effective gas exchange and nutrient distribution. The growth cycle continued until the stationary phase, which is typical after 21 days. To maintain the culture, the depleted medium was replaced aseptically with fresh media.^123^

#### 2.2.7 Harvesting of microalgae

Microalgal cultures were harvested during the exponential growth phase, typically between days 14 and 16 of incubation, when the optical density at 680 nm (OD_680_) reached approximately 1.6. Harvesting at this stage assured that the biomass was metabolically active and ready for further analysis. Algal cells were isolated from the culture media by initial gravity sedimentation, followed by centrifugation at 7000 rpm for 15 minutes at room temperature.^124^ The harvested biomass was either utilized directly or stored at 4 °C for subsequent use.

#### 2.2.8 Promised strain selection

Strain exhibiting the highest growth rate was selected utilizing three complementary methods: optical density (OD) measurements at 680 nm, direct cell counts (cells per mL) using a Neubauer-improved hemocytometer under a light microscope at 400× total magnification, and gravimetric analysis for dry biomass.^125–127^ For biomass quantification, 20 ml of culture was taken and placed in preweighed glass tubes. The Samples were centrifuged at 4000 rpm for 15 minutes to separate the biomass. The supernatant was collected, and the pellet containing tubes were dried in a hot air oven set to 105 °C for 24 hours. The tubes were subsequently reweighed to establish their dry weight. The biomass concentration was estimated by subtracting the end and beginning tube weights. Measurements were taken in triplicate and findings were presented as mean ± SD.^128,129^ Selecting the highest growth rate strain allows focusing on microalgae with maximal biomass and biosorption potential, directly supporting the research objectives.

### 2.3 Preparation of cadmium solutions

A primary stock solution of cadmium (1000 mg L^−1^) was prepared by accurately dissolving cadmium sulfate octahydrate (CdSO_4_·8H_2_O(in ultrapure deionized water. A series of working solutions at concentrations of 10, 20, 50, and 80 mg L^−1^ were prepared before each experimental run.

### 2.4 Collection of natural industrial wastewater samples

Real industrial wastewater samples were collected from two different manufacturing plants in the Kom Abu Rady Industrial Zone, Beni Suef Governorate, Egypt. The sampling area's geographic coordinates are latitude 28°45′ to 29°25′ N and longitude 30°45′ to 31°15′E. All samples were collected in sterile containers, transported on ice, and maintained at 4 °C until further testing to ensure their physicochemical integrity.

### 2.5 Preparation of biosorbent using different immobilization techniques

#### 2.5.1 Preparation of control alginate beads without microalgae

Sodium alginate gel was prepared by dissolving 2 g sodium alginate powder in 100 ml of distilled water and gently spin it overnight, to achieve complete dissolution and homogeneity. To make uniform spherical beads, a viscous solution was transferred to a peristaltic pump and extruded dropwise (1 drop per sec) from a height of 2.5 cm into a gently agitated 2% (w/v) CaCl_2_ solution. Each milliliter of gel solution yielded around 40 beads with an average diameter of around 4.0 mm. To complete crosslinking, the beads were gently spun in CaCl_2_ solution for a further hour. The beads were then carefully washed with distilled water to eliminate any remaining calcium ions and kept in a sterile condition at 4 °C until use.^130^

#### 2.5.2 Preparation of algal beads

Following two weeks of culture, microalgal biomass in the exponential growth phase (approximately 2.5 × 10^8^ cells per mL and OD_680_ = 1.6) was harvested using centrifugation at 3000 rpm for 10 minutes. The supernatant was discarded, and the algal pellet was thoroughly washed with deionized water before being centrifuged one more time to remove any remaining medium components.^131,132^ To achieve homogeneous cell dispersion, mix 2.3 g of concentrated wet algal biomass with 16 ml of 2% (w/v) sodium alginate solution and spin for 15 minutes. The algal alginate mixture was extruded dropwise into a 2% (w/v) CaCl_2_ solution using a peristaltic pump, resulting in homogenous beads with an average diameter of ∼4.0 mm by ionic gelation. The algal beads were left to harden for an hour at 4 °C before being thoroughly washed with distilled water. The prepared microalgal beads were refrigerated before usage.^133^

#### 2.5.3 Preparation of alginate thin film (without microalgae) as a control

To prepare the alginate thin film, filter paper soaked in 5% CaCl_2_ solution was placed in 2 Petri dish (8 ml). A fixed circle of (8 ml of 2% (w/v) alginate gel) was poured over the filter paper and covered with another filter paper pre-wetted with 5% CaCl_2_. The assembly was incubated at 4 °C for 15 minutes to solidify. The produced thin film was then transferred to a Petri plate with 5% CaCl_2_ and allowed for another 15 minutes. Finally, the alginate thin film was rinsed with bi-distilled water. The control films were cut into homogeneous cubes (4 mm × 4 mm × 1 mm) and stored at 4 °C for future use. This approach was implemented and modified in line with Shaaban *et al.*^134^

#### 2.5.4 Preparation of algal thin film

After two weeks of cultivation, microalgal biomass in the exponential growth phase (about 2.5 × 10^8^ cells per mL and OD_680_ = 1.6) was harvested using centrifugation at 3000 rpm for 10 minutes. The supernatant was discarded, and the algal pellet was carefully rinsed in deionized water before being centrifuged again to eliminate any leftover medium components. To obtain homogenous cell dispersion, combine (1.15 g of concentrated wet algal biomass) with (8 ml of 2% (w/v) sodium alginate gel) and spin for 15 minutes. Filter paper Soaked with 5% CaCl2 was placed on (Petri-dish 8 ml). The alginate algal gel was poured on filter paper with a fixed circle, then covered with another wetted filter paper with 5% CaCl2. The setup was kept at 4 °C for 15 minutes to facilitate crosslinking and thin film algal formation. The agal thin film was moved to a second Petri dish with 5% CaCl_2_ for additional 15 minutes to finish the gelation process. The algal thin film was rinsed with bi-distilled water. The algal thin films were cut into homogeneous cubes (4 mm × 4 mm × 1 mm) and stored at 4 °C for future use. The method was modified from the methodology provided by.^134^ Adjustments were implemented for gel volume, CaCl_2_ concentration, film size, and crosslinking conditions to enhance film integrity and consistent biomass incorporation.

### 2.6 The comparative between different techniques of biosorbent on Cd(ii) biosorption

The experiment was carried out by utilizing different biosorbent techniques: wet biomass, alginate immobilized algal beads and novel alginate algal thin film), all different forms of biosorbent contain the same microalgal dose (2.3 gm) in (16 ml) of sodium alginate gel (0.1 M)/50 ml of (10 ppm) concentration of Cd concentration at (25 °C) and (pH 7). Sodium alginate beads and sodium alginate thin films without microalgae strain were employed as controls.

### 2.7 Evaluation of factors affecting Cd(ii) biosorption using algal thin films

#### 2.7.1 Effect of pH on Cd(ii) biosorption using algal thin films

The experiment was employed (2.3 gm) microalgal dose in (16 ml) of sodium alginate gel (0.1 M)/(10 ppm) concentration of Cd solution at (25 °C), with varying pH from 3 to 9. Adjusting pH with (1N) HCl, or (1N) NaOH as needed.

#### 2.7.2 Effect of temperature on Cd(ii) biosorption using algal thin films

We utilized (2.3 gm) of microalgal dose in (16 ml) of sodium alginate gel (0.1 M)/(50 ml) of (10 ppm) concentration of Cd solution at (pH 7) with varying temperature levels (25, 30, 40, and 60 °C).

#### 2.7.3 Effect of microalgal dose on Cd(ii) biosorption using algal thin films

We employed different microalgal doses (2.3 gm, 1.5gm, 0.75gm and 0.30gm) in (16 ml) of sodium alginate gel (0.1 M)/50 ml of (10 ppm) concentration of Cd solution at (25 °C) and (pH 7).

#### 2.7.4 Effect of initial cadmium(ii) concentration on its biosorption using algal thin films

The study was conducted at different concentration of Cd solution (10, 20, 50, 80) ppm, using (2.3 gm) of microalgal dose in 16 ml of sodium alginate gel (0.1 M)/50 ml of Cd solution at 25 °C and pH 7.

#### 2.7.5 Effect of contact time on Cd(ii) biosorption using algal thin films

The experiment was conducted at different time contact (3 h, 6 h, 12 h, 18 h, 24 h), using (2.3 gm microalgal dose in (16 ml) of sodium alginate gel (0.1 M)/(50 ml) of (10 ppm) concentration of Cd solution at (25 °C) and (pH 7).

#### 2.8 Evaluation of Cd^2+^ removal efficiency by alginate, algal thin films and their controls in real industrial wastewater

The experiment was carried out by using (2.3 gm) microalgal dose in (16 ml) of sodium alginate gel (0.1 M)/(50 ml) of wastewater sample had 0.4 ppm and wastewater sample had 2.4 ppm concentration of Cd ions at (25 °C) and (pH 7). Alginate thin films were used as controls for this experiment.

### 2.9 Determination of cadmium removal efficiency (*R*%)

The determination of Cd concentrations in aqueous solution and in real industrial wastewater before and after the biotreatment was measured by using (the Agilent 4200 MP-AES) at Institute of Global Health and Human Ecology, School of Sciences and Engineering, The American University in Cairo. Natural wastewater samples didn't need digestion because they were filtered and diluted 10 times. Calibration was done with standard solution of Cd element and was prepared in 2% nitric acid. The Cd(ii) removal percent (*R*%) was calculated from the eqn (1).

1 (*R*%) = (*C*_b_ − *C*_a_)/*C*_b_ × 100

where: *C*_b_: concentration of *C*_d_ before treatment *C*_a_: concentration of *C*_d_ after treatment.

### 2.10 Statistical analysis

All experimental measurements were performed in triplicate, and the results are reported as means ± standard error (SE). Statistical significance was assessed using one-way analyses of variance^135^ and determined significant differences among means at a confidence level of *p* < 0.05. All statistical computations and graphical representations were conducted using GraphPad Prism software (version 8.0.2, GraphPad Software Inc., USA).

### 2.11 Characterization of thin films (hydrogels)

#### 2.11.1 Fourier-transform infrared analysis (FTIR)

Fourier-transform infrared spectroscopy was employed to investigate the functional groups present in dry alginate and *Chlorella sorokiniana HMY-C* thin films (before and after cadmium biosorption) with a VERTEX 70v FT-IR spectrometer (Bruker, Germany) at National Center for Radiation Research and Technology, Egyptian Atomic Energy Authority, Cairo, Egypt.

#### 2.11.2 X-ray diffraction analysis (XRD) analysis

*Chlorella sorokiniana HMY-C* thin films following cadmium biosorption were investigated using XRD using (SHIMADZU XRD 6000 X-RAY DIFFRACTOMETER (XRD)) at the National Center for Radiation Research and Technology, Egyptian Atomic Energy Authority, Cairo, Egypt.

#### 2.11.3 The zeta potential determination

The surface charge characteristics of algal thin films before biosorption of cadmium were determined by conducting zeta potential measurements across a pH 6 by using (ZEISS-EVO 15-UK) at National Center for Radiation Research and Technology, Egyptian Atomic Energy Authority, Cairo, Egypt.

#### 2.11.4 Scanning electron microscope and energy-dispersive X-ray analysis (EDX) Studies

The examination of dry *Chlorella sorokiniana HMY-C* thin films, before and after cadmium biosorption, was examined utilizing SEM (ZEISS-EVO 15-UK) and EDX analysis (ZEISS Smart EDX) attached to SEM at the National Center for Radiation Research and Technology, Egyptian Atomic Energy Authority in Cairo, Egypt.

### 2.12 Essential analysis of the biosorption mechanism study

#### 2.12.1 Kinetic study

Three distinct models were employed to characterize the sorption kinetics of Cadmium biosorption by *Chlorella sorokiniana HMY-C* thin films.

(A) The pseudo-first-order model, eqn (2):

2 Log(*q*_e_ − *q*_*t*_) = log *q*_*e*_ − *k* × *t*/2.303 (*q*_*t*_)

Representing the amount adsorbed at time (*t*),^22^ as the amount adsorbed at equilibrium (mg g^−1^), and (*k*_1_) as the rate constant for pseudo-first-order adsorption hour^−1^).^134^

(B) The pseudo-second-order model, eqn (3):

3 *t/q*_*t*_ = 1/(*k*_2_*q*_e_^2^) + (1/*q*_e_) × *t*

where (ref. 22) and (*q*_*t*_) represent the adsorption capacities at equilibrium and time (*t*) (mg g^−1^), respectively, while (k2) is the rate constant of the pseudo-second-order sorption (g mg^−1^ hour). When plotting (t/qt) *versus* (t), a linear plot is obtained.^134^ The values of the adsorption parameters qe and k2 can be determined from the slope and intercept of the plot, respectively.

(C) The intraparticle diffusion model, eqn (4):

4 *q*_*t*_ = *k*_*it*_^(0.5)^ + 1

It is utilized to understand the mechanism and rate-controlling steps within the kinetics of biosorption. It offers essential information regarding the process.^136^ Where (*k*_i_) is the intraparticle diffusion rate constant and (*I*) is the intercept, the value of *k*_i_ is determined from the slope of the plot *q*_*t*_*vs. t*^0.5^.

#### 2.12.2 Adsorption isotherms

Numerous models are available in scientific literature to represent adsorption isotherms.^137^ This study specifically focused on four frequently utilized models, were examined to assess the efficiency of *Chlorella sorokiniana HMY-C* thin films in biosorption of Cd ions through the application of Langmuir, Freundlich, Sips and Dubinin–Radushkevich models.

(A) The Langmuir isotherm:

It characterizes even adsorption without the movement of adsorbate across a surface with a limited number of adsorption sites, which are for monolayer adsorption. To achieve equilibrium, the linear version of the Langmuir isotherm model was used to analyze the experimental data as, eqn (5).

5 *C*_e_/*q*_e_ = (1/(*b* × *q*_m_)) + (*C*_e_/*q*_m_)

The adsorbate concentration at equilibrium is denoted as (*C*_e_) in units of mg L^−1^, while the maximum adsorption capacity is represented by (*q*_m_) in units of mg g^−1^, and the Langmuir constant is denoted as (*b*)^138^eqn (6).

6 *R*_L_ = (1/(1 + *b* × *C*_i_))

The initial solute concentration is denoted by (*C*_i_), and (*b*) represents Langmuir's adsorption constant (L mg^−1^). The (*R*_L_) value indicates whether the adsorption is unfavorable (*R*_L_ > 1), linear (*R*_L_ = 1), favorable (0 < *R*_L_ < 1), or irreversible (*R*_L_ = 0).^139,140^

(B) Freundlich isotherm model, eqn (7):

7 Ln *q*_e_ = ln *K*_F_ + (1/*n*) × ln *C*_e_

The Freundlich isotherm serves as an empirical model for heavy metal ion adsorption. Where (ref. 22) is the amount of metal ion adsorbed onto the surface of algal biomass at equilibrium (mg g^−1^) and (*C*_e_) is the equilibrium concentration of the adsorbate (mg L^−1^).^141,142^ The adsorption constant (*K*_F_) signifies the adsorption capacity, while (1/*n*) represents the adsorption intensity, with (*n*) being dependent on the adsorbate and adsorbent. Any value of (*n*) is above one favors the adsorption process.

(C) Sips model, eqn (8):8
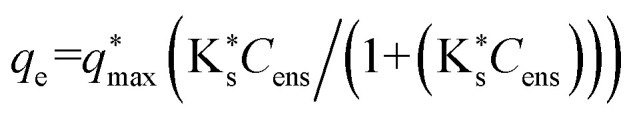
where (ref. 22) is the amount of metal ion adsorbed onto the surface of algal biomass at equilibrium (mg g^−1^) and (*C*_e_) is the equilibrium concentration of the adsorbate (mg L^−1^), The maximum amount of cadmium sorbed by the microalgae is represented by (*q*_max_) (mg g^−1^). The adsorption constant KDR signifies the adsorption capacity.^143^

(D) Dubinin–Radushkevich model, eqn (9):9
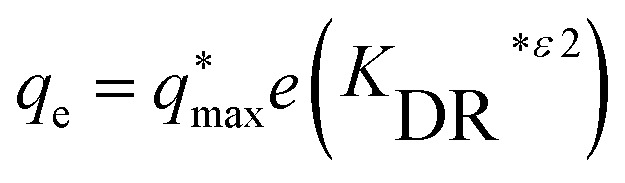


The maximum amount of cadmium sorbed by the microalgae is represented by (*q*_max_) (mg g^−1^), while (*C*_e_) (mg L^−1^) stands for the cadmium concentration at equilibrium. The constant related to the isotherm model is denoted as (*K*_DR_) with *R* representing the kinetic gas constant (8.314 J mol^−1^ K^−1^and *T* (K) indicating the temperature of the system.^144,145^

Linear form of D. R model, eqn (10):

10 Ln *q*_e_ = ln *q*_m_ − *β ε*^2^

where, (*β*) (mol^2^ kJ^−2^) related to constant of adsorption energy, (*ε*) is the Polanyi potential could be determined from, eqn (11).

11 *ε* = *RT* × ln(1 + (1/*C*_e_))

The mean free energy (kJ mol^−1^) could be calculated using, eqn (12):

12 *E* = 1/√2*β*

The (*E*) value indicates whether the nature of biosorption process is physical adsorption or chemisorption process.

## 3 Results and discussion

### 3.1 Characterization of isolated microalgal strains

#### 3.1.1 Purification

**Fig. 2:**
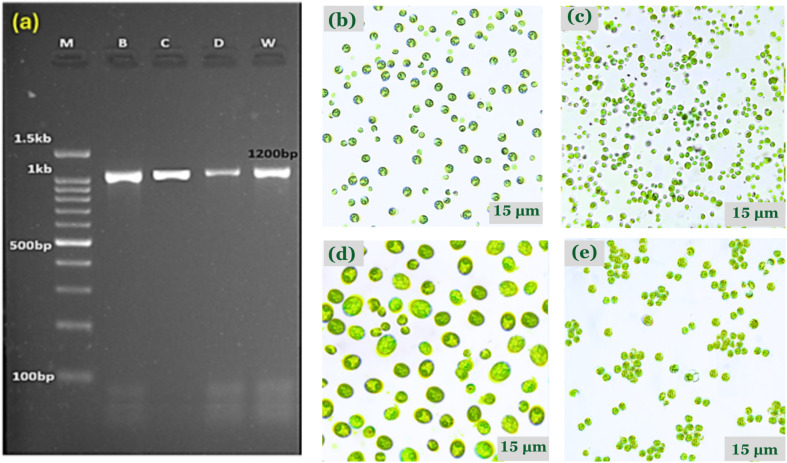
(a) Gel electrophoresis of PCR products of 18S gene on 1.5% agarose gel of four samples: M: DNA marker, B (sample 1), C (sample 2), D (sample 3), and W (sample 4). (b) Microscopy image ×40 for *Parachlorella kessleri* (c) Microscopy image ×40 for *Chlorella sorokiniana*, (d) Microscopy image ×40 for *Scenedesmus vacuolatus*, (e) Microscopy image ×40 for *Auxenochlorella pyrenoidosa*.

To reduce microbial effects, a pre-isolation technique using enrichment and density-based centrifugation was developed. The samples were centrifuged at 4000×*g* for 10 minutes to separate microalgal cells due to their density.^146–148^ The lighter bacterial cells stayed in the supernatant and were discarded. Four distinct green microalgal strains were successfully isolated and purified using aseptic techniques as shown in Fig. 2. To test purity, aliquots of each culture were inoculated in nutrient agar plates and incubated for 48 hours at 28 °C. There were no bacterial colonies detected under these conditions, indicating that heterotrophic contaminants had been eradicated. The rigorous purification technique assured that any future research, whether on growth dynamics, pollutant absorption, or metabolic profile, could be ascribed only to the physiological activity of the microalgal strains. This methodological soundness improves the dataset's repeatability, interpretability, and scientific integrity.^149,150^

#### 3.1.2 Molecular identification

Molecular identification of the purified green microalgal strains was achieved using PCR amplification of the 18S rRNA gene with species conserved primers (DSs and DPs). To confirm effective amplification, the amplified products were run on a 1.5% agarose gel, Fig. 2. The purified PCR products were sequenced and evaluated using the NCBI Basic Local Alignment Search Tool (BLAST). The BLAST findings showed high sequence similarity (≥99%) to recognized microalgal species, assisting precise species identification. The strains were subsequently submitted into the GenBank database and assigned accession numbers as shown in Table 3.

**Table 3:** The four pure green unialgal strains registered in GenBank (accessions and link)^a^

Sample	Submission	Accession number	Strain identification
B	SUB14206830 Seq1	PP273965	*Parachlorella kessleri HMYA-B (https://www.ncbi.nlm.nih.gov/nuccore/PP273965)*
C	SUB14206830 Seq2	PP273966	*Chlorella sorokiniana HMYA-C (https://www.ncbi.nlm.nih.gov/nuccore/PP273966)*
D	SUB14206830 Seq3	PP273967	*Scenedesmus vacuolatus HMYA-D* (*https://www.ncbi.nlm.nih.gov/nuccore/PP273967)*
W	SUB14206830 Seq4	PP273968	*Auxenochlorella pyrenoidosa HMYA-W* (*https://www.ncbi.nlm.nih.gov/nuccore/PP273968)*

a SUB: refers to the GenBank submission number; Seq: refers to the sequence code. Accession numbers are officially registered in the NCBI GenBank database.

**Fig. 3:**
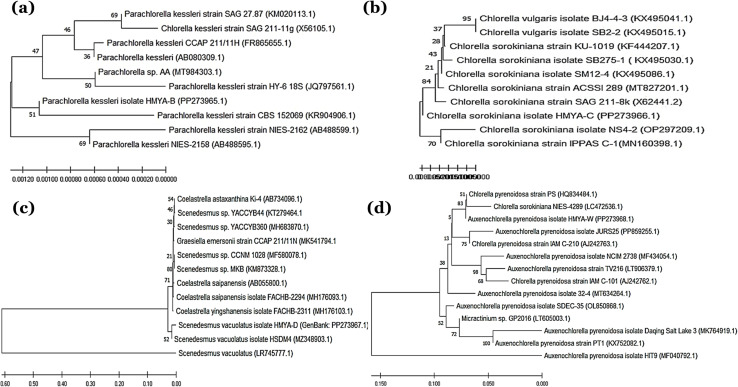
(a) The phylogenetic tree of B strain depicted the evolutionary relationship between the isolated strain (B) and similar microalgal species, proving its taxonomic identification, constructed using the powerful NCBI Blast tool based on The 18S rRNA sequence. (b) The phylogenetic tree of C strain depicted the evolutionary relationship between the isolated strain (d) and similar microalgal species, proving its taxonomic identification, constructed using the powerful NCBI Blast tool based on The 18S rRNA sequence. (c) The phylogenetic tree of D strain depicted the evolutionary relationship between the isolated strain (D) and similar microalgal species, proving its taxonomic identification, constructed using the powerful NCBI Blast tool based on The 18S rRNA sequence. (d) The phylogenetic tree of W strain depicted the evolutionary relationship between the isolated strain (W) and similar microalgal species, proving its taxonomic identification, constructed using the powerful NCBI Blast tool based on The 18S rRNA sequence.

To validate the molecular identification, phylogenetic analysis was performed using MEGA11 software (Molecular Evolutionary Genetics Analysis version 11) and the Maximum Likelihood technique with 1000 bootstrap replicates.^121^ Phylogenetic trees demonstrated the evolutionary relationships of the isolated strains with their closest known relatives, Fig. 3.

#### 3.1.3 Growth assessment and promising strain selection

**Fig. 4:**
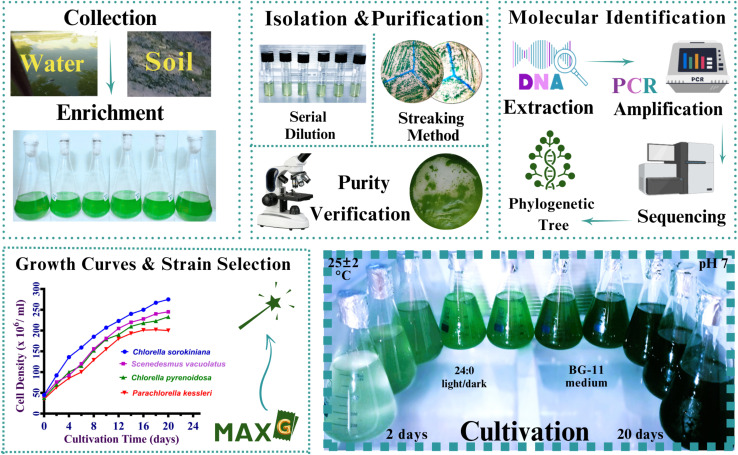
The workflow form samples collection to promising strain selection. Samples of environmental water and soil were obtained, and enrichment was performed. Microalgal strains were isolated and purified using serial dilution and streaking procedures, with culture purity verified under the microscope. DNA extraction, PCR amplification, sequencing, and the creation of a phylogenetic tree were all used to corroborate taxonomic positioning. Cultivation was optimized in BG-11 medium (pH 7), with continuous illumination (24 : 0 light/dark cycle) at 25 ± 2 °C for 20 days. After comparing growth rates, *Chlorella sorokiniana HMYA-C* was identified as the most promising strain.

Four microalgal strains were cultivated under standardized conditions using two distinct growth media: BG-11 and Wuxal. The cultures were maintained under continuous white light (24 : 0 h light/dark photoperiod) at a controlled pH of 7 and temperature of 25 ± 2 °C, Fig. 4. Suboptimal temperatures (below 16 °C) might inhibit algal growth, whereas temperatures above 35 °C can be lethal to numerous algal species.^119^ pH plays a pivotal role in metabolic activity and nutritional availability in culture.^151–153^ Among environmental parameters, light intensity is the most important predictor of microalgal development and biomass accumulation. Continuous white light was shown to stimulate the growth of *Haematococcus lacustris* more than intermittent blue or red light (12 : 12 h cycle).^154^ Furthermore, spectral quality and light exposure duration have a significant influence on the biochemical profile of algal biomass due to their impact photosynthetic efficiency.^155^ BG-11 medium, also referred to as Blue green 11 medium, is a widely adopted synthetic medium for a broad range of species and is particularly effective for freshwater strains. It is also frequently employed in lipid synthesis studies due to its well-balanced nutritional profile.^156^ In comparison, Wuxal is a widely available, broad-spectrum liquid fertilizer (N 8%, P_2_O_5_ 8%, K_2_O 6%, Mn 0.012%, Fe 0.02%, B 0.01%, Cu 0.004%, Zn 0.004%) that was used in this work as a cost-effective and simplified nutritional medium.^157^ All algal strains demonstrated high growth rate on Blue green 11 than Wuxal medium.

**Fig. 5:**
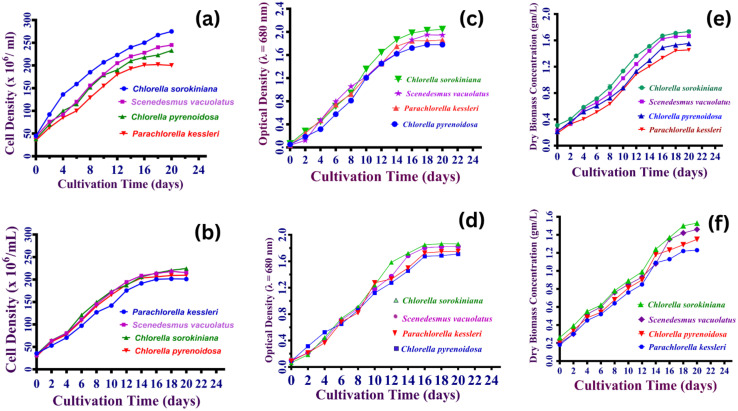
(a) The growth curve of microalgae strains cultivated in BG11 medium, measured as cell density (cells per mL) over time under optimized conditions: pH 7, continuous illumination (24 : 0 light: dark cycle), and a temperature of 25 ± 2 °C for 20 days. (b) The growth curve of microalgae strains cultivated in Wuxal medium, measured as cell density (cells per mL) over time under optimized conditions: pH 7, continuous illumination (24 : 0 light: dark cycle), and a temperature of 25 ± 2 °C for 20 days. (c) The growth curve of microalgae strains cultivated in BG11 medium monitored as optical density (680 nm) over time under optimized conditions: pH 7, continuous illumination (24 : 0 light: dark cycle), and a temperature of 25 ± 2 °C for 20 days. (d) The growth curve of microalgae strains cultivated in Wuxal medium, monitored as optical density (680 nm) over time under optimized conditions: pH 7, continuous illumination (24 : 0 light: dark cycle), and a temperature of 25 ± 2 °C for 20 days. (e) The growth curve of microalgae strains cultivated in BG11 medium expressed as dry weight (g L^−1^) over time under optimized conditions: pH 7, continuous illumination (24 : 0 light: dark cycle), and a temperature of 25 ± 2 °C for 20 days. (f) The growth curve of microalgae strains cultivated in Wuxal medium, expressed as dry weight (g L^−1^) over time under optimized conditions: pH 7, continuous illumination (24 : 0 light: dark cycle), and a temperature of 25 ± 2 °C for 20 days.

Microalgae species demonstrated rapid growth and high nutrient absorption efficiency, indicating their potential for efficient wastewater treatment.^158^ In this investigation, the development of four algal strains was observed under controlled settings for 18 days. Measurements were taken every two days, in triplicate, using three complementary analytical methods: cell density enumeration using a hemocytometer, based on direct microscopic counting;^127^ optical density (OD) measurements at 680 nm to estimate chlorophyll-containing biomass;^159^ and (3) quantification of dry biomass concentration, determined by gravimetric analysis of dried algal biomass per liter of culture, Fig. 5,.^160^ Cell density measurements were shown to be the most reliable of these approaches, providing a direct and quantitative indication of viable cell populations.^161^ In contrast, OD measurements may be influenced by the presence of dissolved or suspended compounds in the culture media, resulting in an overestimation or underestimating of biomass.^162^ Similarly, dry weight measurements are subject to inaccuracies caused by poor biomass recovery or cell lysis during the harvesting process, especially if the approach is not fully optimized. Among the four microalgal strains, *Chlorella sorokiniana HMYA-C* exhibited superior growth rate, achieving the maximum cell density, optical density, and dry biomass concentration on day 18, as shown in Fig. 5. Unicellular green microalga *Chlorella sorokiniana HMYA-C* excellent growth performance in nutrient-rich environments shows a higher physiological capability for nutrient absorption, positioning it as a strong candidate for advanced wastewater treatment.

### 3.2 The comparative between the Cd biosorption efficiency of different form of biosorbent

**Fig. 6:**
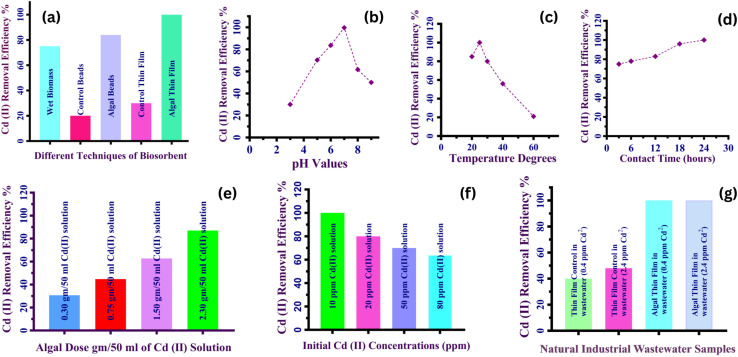
(a) Differentiation between the biosorption efficiency of different techniques of *Chlorella sorokiniana* biosorbent for removal of cadmium from aqueous solution with the same condition, (pH 7, temperature 25 °C, 2.3 g/16 ml sodium alginate gel/50 ml of 10 ppm cd concentration), demonstrated that the microalgae in the form of thin film)rectangular sheets(outperform typical spherical alginate beads and wet biomass in cadmium removal efficiency. (b) Impact of pH on (Cd(ii) biosorption using (2.3 gm) of microalgal dose in (16 ml) of sodium alginate gel/(50 ml) of (10 ppm) concentration of Cd solution at (25 °C), with varying pH from 3 to 9, revealing that pH 7 is the optimal pH for Cd(ii) removal utilizing *Chlorella sorokiniana* thin film. (c) The Impact of Temperature on the biosorption of Cd(ii) utilizing (2.3 gm) biomass in (16 ml) of sodium alginate gel/(50 ml) of (10 ppm) concentration of Cd solution at (pH 7) with varying temperature levels (25, 30, 40, and 60 °C, indicating that the temperature at 25 °C producing the greatest mean removal% of all temperatures examined. (d) The impact of the biosorbent dose on Cd removal efficiency using different microalgal dose (2.3gm, 1.5gm, 0.75gm and 0.30gm) in (16 ml) of sodium alginate gel/50 ml of (10 ppm) concentration of Cd solution at (25 °C) and (pH 7). (e) The impact of the initial metal concentration on the biosorption of Cd(ii) using different concentration of Cd solution (10, 20, 50, 80) ppm, (2.3 gm) biomass in (16 ml) of sodium alginate gel/50 ml of Cd solution at (25 °C) and (pH 7). (f) The impact of contact time on the biosorption of Cd(ii) at different contact times (3, 6, 12, 18, 24) h, (2.3 gm) biomass in (16 ml) of sodium alginate gel/50 ml of Cd solution at (25 °C) and (pH 7). (g) Cadmium(ii) removal efficiency % utilizing algal thin films (hydrogel) and their controls in natural industrial wastewater indicating that 100% removal efficiency of cadmium in two different samples of real industrial wastewater (0.4 ppm Cd) and (2.4 ppm Cd).

*Chlorella sorokiniana HMYA-C* biosorbent was employed in three different configuration (suspended wet biomass, an immobilized beads and a novel immobilized thin film (hydrogel)) for removal of cadmium from aqueous solution with the same condition pH 7, temperature 25 °C, (2.3 gm live concentrated harvested algal biomass)/16 ml sodium alginate gel (0.1 M)/50 ml of 10 ppm cd concentration). Algal biosorbents in the form of thin film)rectangular sheets(outperform typical spherical alginate beads and wet biomass in Cadmium removal efficiency, as shown in Fig. 6a. The physical structure of the biosorbent has a considerable impact on both the biosorption process and efficiency.^163^ Thin films have more active sites for cadmium adsorption than beads with the same biomass content because of their longer, planar shape, which enhances their effective surface area. Furthermore, the thin film morphology shows rougher and more porous, producing many diffusion paths, promoting deeper cadmium trapping in the matrix and accelerating intraparticle diffusion. The finding is consistent with previous research that has established a direct association between surface roughness, specific surface area.^164^ In contrast, the immobilized thin film structure utilized in this study significantly increases biosorption efficiency. A one-way ANOVA (*F*(4, 10) = 2072, *p* < 0.0001, *R*^2^ = 0.9988) show that the treatment groups accounted for virtually all variation in the response. These findings indicate the algal thin-film system's superior efficacy, highlighting its improved functionality and tremendous potential as an effective and sustainable biosorbent for cadmium removal. Compared to suspended-cell cultures, the immobilized design showed significantly better metal uptake, owing to its higher surface roughness, bigger specific surface area, and increased stability. Conventional free-cell systems frequently face issues such as difficult harvesting, decreased metabolic activity under metal stress, and environmental concerns due to potential release into natural ecosystems particularly when genetically modified strains are used.^165,166^ In contrast, immobilization within a polymeric matrix provides a strong, recyclable platform that sustains cell viability and activity by allowing for nutrition and gas exchange while shielding cells from harsh environmental variations.^135^ This arrangement reduces biomass washout, improves tolerance to hazardous metals, and reduces contamination hazards. Among immobilized systems, the thin-film design outperformed bead-based matrices by removing internal diffusion constraints, enabling more effective exploitation of active sites, and facilitating improved mass transfer. Furthermore, its planar construction supports even light dispersion and efficient gas exchange, hence maintaining photosynthetic performance and total biosorption efficiency.^167^ These features emphasize the thin-film immobilization system as a technically advanced, scalable, and safe solution for ongoing industrial bioremediation of heavy metals like cadmium. Recent studies have highlighted the superior performance of thin-film hydrogels as advanced platforms for bioremediation, owing to their high surface-to-volume ratio, enhanced mass transfer, and improved structural stability. For example, immobilization in a thin film shape was significantly more effective than immobilization in beads for Co(ii) elimination in bacteria and fungus.^134^ Collectively, these findings highlight the critical significance of thin-film hydrogel systems as next-generation bioremediation materials. Their unique combination of mechanical stability, reusability, and improved mass transfer makes them an environmentally safe, cost-effective, and highly efficient solution for the continuous removal of hazardous metals from wastewater.

### 3.3 Factors affecting cadmium biosorption

Following characterization and selection of the promising microalgal strain, we evaluated the effects of various operational factors on its cadmium biosorption performance.

#### 3.3.1 The influence of initial pH on the biosorption behavior of Cd(ii) using *Chlorella sorokiniana HMYA-C* thin films

The pH of the solution is critical to the biosorption process because it affects metal ion solubility, speciation, toxicity, and the ionization state of functional groups on the surface.^168^ In this study, The influence of initial pH on Cd(ii) removal efficiency was examined throughout a pH range of 3 to 9, with three replicates per condition. As shown in Fig. 6b, the removal efficiency increased progressively from 25% at pH 3 to 100% at pH 7. This spike is caused by enhanced deprotonation of functional groups on the algal surface, which increases the availability of negatively charged sites capable of binding positively charged Cd(ii) ions.^169^ These findings are consistent with previous study, which identified pH 7 as the best value for heavy metal biosorption using algal biomass.^39^ In acidic conditions (pH = 3), H^+^ ions compete with Cd^2+^ for binding sites on the biosorbent surface. This proton saturation interferes with the coordination of acidic functional groups (*e.g.*, carboxyl, phosphate) with metal ions, interrupting the biosorption process and reducing efficiency.^164^ Above pH 7, removal efficacy decreases due to the production of insoluble Cd(OH)_2_ precipitates, limiting the availability of free Cd^2+^ in solution.^170^ A one-way ANOVA revealed significant pH differences (F(1.305, 6.526) = 27.98, *P* = 0.0010, *R*^2^ = 0.8484). This indicates that pH fluctuations contribute to 84.84% of the difference in removal efficiency, highlighting the role of pH in controlling metal ion bioavailability and surface charge interaction between the biosorbent and metal ions.

#### 3.3.2 The influence of temperature on the biosorption behavior of Cd(ii) using *Chlorella sorokiniana HMYA-C* thin films

The impact of temperature on the biosorption efficacy of Cd(ii) by algal thin film was systematically investigated at temperatures of (25 °C, 30 °C, 40 °C, and 60 °C). The results in Fig. 6c indicated that the greatest biosorption efficiency of 100% was consistently achieved at 25 °C, this agreement with.^171^ Efficiency decreased gradually at both low and high temperatures, with the lowest value obtained at 60 °C. At 25 °C, the biosorption process benefits favorable thermodynamic and kinetic conditions, which optimize the interaction of Cd(ii) ions with the functional groups on the biosorbent surface. Higher temperatures may promote denaturation or instability of the biosorbent's active binding sites, resulting in lower biosorption effectiveness. This is consistent with prior research, which indicates that greater temperatures promote enhanced solubility of metal ions, decreasing the ion–adsorbent interaction.^172^ The influence of temperature on algal thin film was found to be consistent with insights from other microalgal systems, where living cells demonstrate variations in metal uptake as a function of temperature, but dead cells respond little to no to temperature swing.^173^ The influence of temperature on metal absorption has also been demonstrated to be strain-dependent.^174^ Temperature had a significant influence on the biosorption efficiency of Cd(ii) utilizing algal thin film, with 25 °C producing the greatest removal% of all temperatures examined. Although the differences did not reach statistical significance using A one-way ANOVA (F(1.001, 4.004) = 2.549, *p* = 0.1856), the high coefficient of determination (*R*^2^ = 0.918) indicates a strong trend toward enhanced removal efficiency at 25 °C. This might suggest that 25 °C provides the optimal kinetic or binding conditions for Cd(ii) absorption. The lack of statistical significance might be attributed to a limited sample size or slight differences between groups. Further research into larger replicas is necessary to validate this observation for enhancing heavy metal biosorption procedures.

#### 3.3.3 The influence of contact time on the Cd(ii) biosorption behavior using *Chlorella sorokiniana HMYA-C* thin films

The study systematically investigated the effect of contact time on the biosorption effectiveness of cadmium using algal thin film, with the objective of determining the best period for maximal removal. Biosorption was assessed at five times (3, 6, 12, 18, and 24 hours), as shown in Fig. 6d. The findings showed a quick initial biosorption phase with around 80% elimination efficacy in the first three hours, followed by a continuous increase to 100% efficiency after 24 hours. This pattern corresponds to the typical three phase biosorption mechanism: an initial phase characterized by physical adsorption onto available binding sites and a large surface area, a slower, diffusion-controlled phase as surface sites are occupied, and final phase indicating equilibrium. A one-way ANOVA analysis (F(1.201, 4.805) = 1906, *p* < 0.0001, *R*^2^ = 0.9979), revealed that contact time significantly affects Cd(ii) removal effectiveness with different contact time treatments accounting for 99.7% of the variance in removal efficacy.

#### 3.3.4 The influence of algal dose on the Cd(ii) biosorption behavior using *Chlorella sorokiniana HMYA-C* thin films

The study investigated how different biosorbent doses affected the removal of cadmium from water solutions. Four different doses were tested: 2.3 g, 1.5 g, 0.75 g, and 0.30 g. As demonstrated in Fig. 6e, the maximum dosage of 2.3 g per 50 ml of Cd solution, along with 16 ml of sodium alginate gel, resulted in the highest removal efficiency. This increase in efficiency is attributed to the increased availability of binding sites, which significantly accelerates the biosorption process, consistent with previous studies.^175^ However, at extremely high biomass levels, removal effectiveness decreased, which might be attributed to biosorbent aggregation. This aggregation may diminish effective surface area and impede access to binding sites by reducing inter-site distances. A one-way ANOVA (*F*(3, 12) = 23.54, *p* < 0.0001, *R*^2^ = 0.8547) revealed that algal dosage significantly impacts Cd(ii) removal efficiency which accounting for almost 85% of the variation in removal efficiency.

#### 3.3.5 The influence of Cd(ii) concentration on the biosorption behavior using *Chlorella sorokiniana HMYA-C* thin films

A series of controlled batch tests were performed to determine the optimal Cd(ii) concentration for maximal biosorption effectiveness, with values ranging from 10 to 80 ppm. As shown in Fig. 6f, the elimination effectiveness was 100% at 10 ppm and then decreased to 64% at 80 ppm. This pattern is related to the number of active binding sites at lower metal ion concentrations, which allows for full biosorption; but, at higher concentrations, site saturation inhibits further adsorption, despite increased biosorption capabilities. A one-way ANOVA analysis (*F*(3, 8) = 472.0, *p* < 0.0001, *R*^2^ = 0.9944) revealed that variations in Cd(ii) concentration explained over 99% of the variance in removal efficiency. These findings show that the initial metal concentration is significant in determining biosorption potential, most likely because higher concentrations offer a stronger driving force for mass transfer.^176^

#### 3.3.6 Biosorption of Cd(ii) from real industrial wastewater using *Chlorella sorokiniana* thin films

Prior to biosorption investigation, real industrial wastewater samples from a textile facility were tested to confirm the presence of different inorganic elements, including calcium, copper (Cu), manganese (Mn), phosphorus (P), and cadmium (Cd). The control studies, which used Ca-alginate thin films without microalgae, yielded significantly lower cadmium removal efficiency, indicating *Chlorella sorokiniana's* critical participation in the biosorption process. A one-way ANOVA (*F*(3,8) = 974.8, *p* < 0.0001, *R*^2^ = 0.9973) confirmed the superiority of the immobilized algal thin film system over the control. Despite the presence of multiple competing ions and organic components in real industrial wastewater, the immobilized *C. sorokiniana* thin films achieved 100% Cd(ii) removal efficiency in two different wastewater samples with initial cadmium concentrations of 0.4 ppm and 2.4 ppm, as shown in Fig. 6g. This outstanding removal performance in complicated effluents demonstrates the algal thin-film matrix's selectivity and high metal-binding affinity. In addition, achieving such high biosorption effectiveness in real industrial wastewater rather than synthetic laboratory media demonstrates the system's practicality. Several previous studies have yielded encouraging results under ideal laboratory circumstances; nevertheless, the presence of various ions, colors, and organic compounds in real wastewater frequently restricts metal absorption. The *C. sorokiniana* thin-film system is resilient, adaptable, and stable under actual environmental conditions, as evidenced by the constant and complete removal observed here. Furthermore, the immobilization of algae cells within the alginate matrix improves biosorption stability while also providing an inherent biosafety advantage. Although the study did not focus on changes in microbial community structure during biosorption, the immobilized *Chlorella sorokiniana HMYA-C* thin-film design was specifically intended to limit microalgal leakage into the surrounding environment. This thin film reduces the possibility of disturbing native microbial communities while also ensuring that biosorption occurs without altering the treated water's natural biological balance. Such design issues are especially important in large-scale or continuous-flow systems, where uncontrolled algal release could result in ecological imbalance or secondary contamination. Thus, the system offers an excellent integration of high removal efficiency, operational stability, and environmental safety as a vital combination for long-term and practical wastewater treatment applications. Overall, these findings represent a significant breakthrough toward scalable, environmentally friendly, and sustainable heavy metal removal technology. The *C. sorokiniana* thin-film system's exhibited power to maintain remarkable performance in challenging industrial effluents highlights its promise as a resilient and selective biosorbent for large-scale wastewater treatment. Future research intends to explain the system's performance in multi-contaminant environments and enhance the operational parameters to maximize the long-term biosorption efficiency and environmental compatibility.

### 3.4 Characterization of thin films (hydrogels)

The thin film was generated by the unicellular green microalga *Chlorella sorokiniana* HMYA-C, which forms a photosynthetic and self-sustaining matrix suitable for metal biosorption.

#### 3.4.1 Fourier-transform infrared analysis (FTIR)

**Fig. 7:**
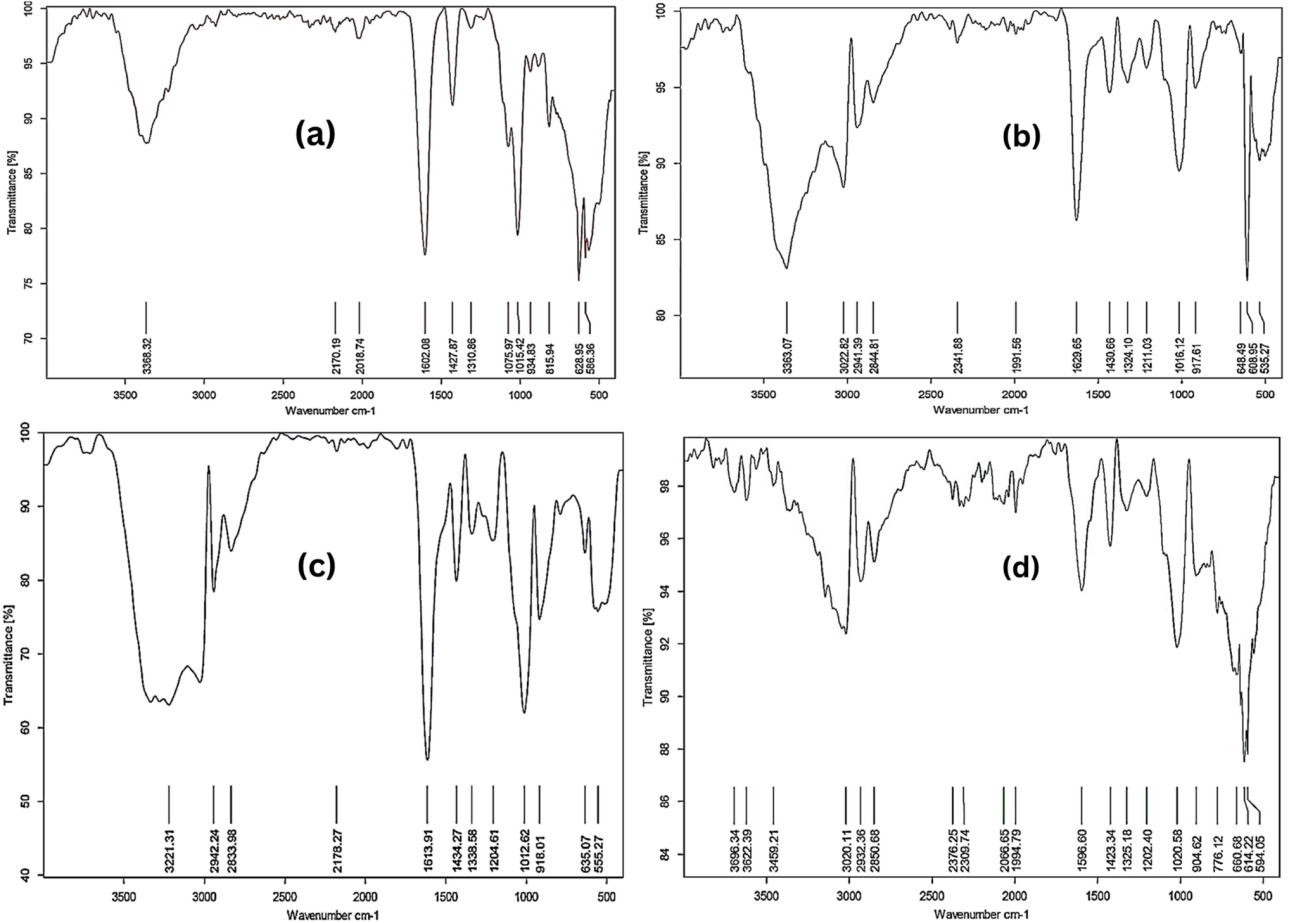
(a) The FTIR spectrum of calcium alginate thin films before Cd(ii) biosorption demonstrating the functional groups on the film surface. (b) The FTIR spectrum of calcium alginate thin films following Cd(ii) biosorption was conducted at 80 ppm Cd^2+^ aqueous solution. Indicating the changes in the functional groups on the film surface. (c) The FTIR spectrum of *Chlorella sorokiniana* thin films prior to Cd(ii) biosorption highlighting the functional groups detected on the algal surface. (d) The FTIR spectra of *Chlorella sorokiniana* thin films following Cd(ii) biosorption was conducted at 80 ppm Cd^2+^ aqueous solution shows changes in functional group vibrations due to metal binding.

FTIR spectroscopy was utilized to analyze the functional groups of alginate and algal thin films (before and after Cd^2+^ biosorption (conducted at 80 ppm Cd^2+^ aqueous solution)), as shown in Fig. 7,Prior to biosorption, the FTIR spectra of algal thin films exhibited distinct absorption bands at 3720–3584 cm^−1^ and 3550–3200 cm^−1^, indicating the O–H stretching vibrations of free and hydrogen-bonded alcohol groups, respectively. Proteins and lipids may be present on the algal surface, as shown by a broad band between 3000-2800 cm^−1^ attributed to N–H stretching and aliphatic C–H vibrations. The algal thin film exhibited chemical complexity, with discrete bands for amine bending (1650–1580 cm^−1^), cyclic alkene C=C stretching (1650–1566 cm^−1^), sulfone groups (1350–1300 cm^−1^), and amine C–N stretching (1250–1020 cm^−1^) as shown in Table 4. The algal thin film FTIR spectra influenced significantly after Cd^2+^ biosorption, showing active binding of several functional groups. The O–H stretching band migrated to lower wavenumbers (3696.34 cm^−1^), while a new peak appeared at 3459.21 cm^−1^, indicating the participation of hydroxyl and amine groups in metal complexation. New bands at 2376.25 and 2309.74 cm^−1^, ascribed to C≡C and O=C=O stretching modes, respectively, reveal the production of unique chemical bonds *via* interactions between Cd^2+^ ions and surface-bound thiocyanate and phosphodiester groups. The ester carbonyl (C=O) band at 1773.00 cm^−1^ vanished following biosorption, highlighting the importance of carbonyl functionalities in cadmium coordination. Shifts in amide-related regions (1325–1202 cm^−1^) and phosphate-related peaks (about 1020 cm^−1^) reveal the role of amide nitrogen and phosphate oxygen in metal binding. Spectral discrepancies between the sulfonyl (S=O) stretching region (1350–1300 cm^−1^) and C–N stretching vibrations suggest a multidentate binding mechanism. Chemisorption of Cd^2+^ onto *C. sorokiniana* thin films mostly occurs *via* hydroxyl, amine, carbonyl, phosphate, and sulfonyl groups, resulting in spectrum alterations and band emergence/disappearance. Specifically, carboxyl and hydroxyl groups mainly participated through ion exchange and electrostatic interactions, while amine and phosphate groups contributed to coordination complexation with Cd^2+^ ions. The substantial, and most likely irreversible, chemical interactions between algae functional groups and cadmium ions highlight *C. sorokiniana HMY-C* thin film superior biosorption efficiency and intriguing potential as an environmentally benign biosorbent for heavy metal removal.

**Table 4:** Functional groups of the calcium alginate thin films and *Chlorella sorokiniana* thin films before and after biosorption of cadmium^a^

G.F Before biosorption *λ* (cm^−1^)	G.F After biosorption *λ* (cm^−1^)	C.F Before biosorption *λ* (cm^−1^)	C.F After biosorption *λ* (cm^−1^)	Bands indicating functional groups
—	—	3720.84	3696.34	(O–H alcohol), polyphenols or polysaccharides^177,178^
			3622.39	
3368.32	3363.07	—	3459.21	(O–H/N–H) carbohydrates, proteins, and lipids^179^
—	3022.82	3023.59	3020.11	(N–H) stretching^180^
—	2941.39	2948.32	2932.36	Aliphatic (C–H/N–H) presence of lipids,.^181,182^
	2844.81	2829.09	2850.68	
—	2341.88	—	2376.25	(C≡C/O=C=O/–S–C≡N) stretching thiocyanate^183,184^
—	—	—	2309.74	
2170.19	—	2125.83	—	
2018.74	—	—	2066.65	(N=C=S/C=C/P=O) isothiocyanate, phytochemicals phosphodiester^185,186^
—	1991.56	—	1994.79	
—	—	1773.00	—	(C=O) esters^187^
—	—	—	1596.6	(–C=C/HC = O/R2 C=O/N–H), cyclic alkene, carbonyl, amines, amides, and some proteins^188,189^
1602.08	1629.65	1611.02	—	
1427.87	1430.66	1436.78	1423.34	C=O/C–H bending^190^
1310.86	1324.1	1332.25	1325.18	(–S=O/C–N–C/N–H), sulfone, stretching of amides from proteins^191–193^
—	1211.03	1211.04	1202.4	(C–N/ –SO_3_/P=O) aromatic compounds, phosphodiester, polysaccharides)^194,195^
1075.97	—	—	—	(P=O bonds in (PO_4_)_3_/C–O–C/C=C bending, –C–O (alcohol)) polysaccharides (The carbohydrate band spectra)^196–198^
1015.42	1016.12	1014.80	1020.58	
934.83	917.61	922.25	904.62	(C=O=P/O–H out-of-plane) (the a-(1,4) glycoside bond, polysaccharides)^199,200^
815.94	—	792.75	776.12	C–H bending/Si–H bending (the carbohydrate band spectra)^201,202^
628.9	648.49	—	660.68	H_2_PO_4_^−^/PO_4_–/N–C=O/Si–O (C–Cl halo compound) (polysaccharides)^203–205^
—	608.95	—	614.22	
586.36	535.27	588.06	594.05	
—	—	510.2	—	

a *λ* (cm^−1^) represents the wavenumber of FTIR absorption bands indicating the presence of functional groups involved in cadmium biosorption. G.F: Alginate thin film C.F: *Chlorella sorokiniana* thin films.

#### 3.4.2 Energy-dispersive X-ray analysis (EDX) studies

**Fig. 8:**
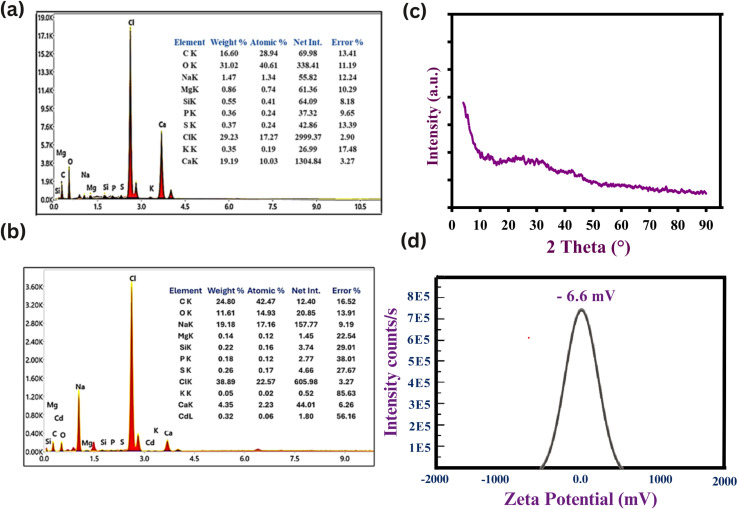
(a) illustrates the EDX spectrum of *Chlorella sorokiniana* thin film before Cd^2+^ biosorption. The spectrum shows the presence of important endogenous elements such as Ca, K, Na, Mg, Cl, P, and S, but no detectable Cd signal, demonstrating the absence of cadmium before exposure. (b) The EDX spectrum of *Chlorella sorokiniana* thin film following Cd^2+^ biosorption, was (conducted at 80 ppm Cd^2+^ aqueous solution). The presence of Cd peaks and decreased Ca, Na, and Mg intensities suggest a cation exchange process where Cd^2+^ displaces lighter cations on the algal surface. (c) The X-ray diffraction (XRD) characterization of the *Chlorella sorokiniana* thin films following Cd^2+^ biosorption (conducted at 80 ppm Cd^2+^ aqueous solution), revealed an amorphous profile with no recognizable crystalline peaks. (d) *Chlorella sorokiniana* thin film showed a mean zeta potential of −6.6 mV at pH 6, demonstrated high colloidal stability and low electrostatic repulsion.

EDX was employed to verify the elemental composition of immobilized *Chlorella sorokiniana* thin films before and after Cd^2+^ biosorption, Fig. 8a and b. Prior to exposure, the EDX spectrum showed significant signals for Ca, K, Mg, Na, Cl, and P, indicating a mineral rich algal surface. Notably, no cadmium signal was discovered, showing that the biomass is pure. After biosorption, a distinct Cd signal developed in the spectrum, and the atomic percentage of calcium decreased significantly from 10.03% to 2.23%, showing ion exchange between Cd^2+^ and endogenous Ca^2+^ ions on the cell wall. This observation suggests a displacement mechanism in which Cd^2+^ replaces lighter metal cations at active sites. Reductions in Na^+^ and Mg^2+^ peaks indicate multivalent cation exchange. These changes, along with the persistent presence of oxygen and carbon rich functional groups, support the involvement of surface bound carboxyl, hydroxyl, and phosphate groups in the biosorption process. The spectroscopic and elemental results indicate that Cd^2+^ is removed by complexation with functional groups and cation exchange at the algal surface.

#### 3.4.3 X-ray diffraction spectrometry (XRD) structural analysis

The X-ray diffraction (XRD) characterization of the *Chlorella sorokiniana* thin films following Cd adsorption (conducted at 80 ppm Cd^2+^ aqueous solution), revealed an amorphous profile with no recognizable crystalline peaks, as shown in Fig. 8c. The smooth baseline and lack of diffraction peaks indicate that no crystalline cadmium phases, such Cd(OH)_2_ or CdCO_3_, were formed during the biosorption process. This study demonstrated that cadmium ions persisted inside the biomass matrix rather than constructing long range crystalline structures, due to the emergence of nanoscale, amorphous complexes with functional groups on the algal surface. The biomass's amorphous shape maintains after adsorption, lending confidence to the mechanism of strong chemical binding rather than physical precipitation of cadmium. Non crystalline immobilization of Cd^2+^ enables chemical desorption with mild acidic or chelating agents, resulting in cadmium recovery and biosorbent recycling. This process prevails in recovering significant heavy metals such as cadmium from different wastewater sources, contributing to environmental sustainability by providing both metal recovery and biosorbent regeneration for further use.

#### 3.4.4 Functional implications of a moderate zeta potential (−6.6 mV) in cadmium biosorption

The zeta potential is a key physicochemical indicator of surface charge and colloidal behavior, having direct consequences for particle stability and adsorption kinetics. The *Chlorella sorokiniana* thin film demonstrated a mean zeta potential of −6.6 mV at pH 6, showing high colloidal stability and low electrostatic repulsion, Fig. 8d. Despite The optimal zeta potential range for removing cations from wastewater is typically −10 mV to −30 mV, with slightly negative to moderately negative charges resulting in effective cation aggregation, flocculation, and adsorption.^206^ However, the precise range depends on the adsorbent material, the kind of cation being removed the system, successfully eliminated cadmium. This study highlights the significance of non-electrostatic systems that collaborate to control biosorption efficiency. Supporting this, EDX elemental analysis revealed substantial reductions in endogenous Ca^2+^, Na^+^, and Mg^2+^ levels, as well as a prominent post adsorption cadmium signal compared to pretreatment samples. The observed alterations suggest that Cd^2+^ displaces lighter cations from functionalized regions on the algal matrix *via* ion exchange. The persistent occurrence of oxygen and phosphorus rich functional groups such as carboxyl, hydroxyl, and phosphate moieties point to ligand specific complexation and hydrogen bonding as adsorption processes. Environmental conditions played a reinforcing impact: Operating at pH 6 maintained cadmium's reactive divalent state (Cd^2+^), while a low ionic the background minimized competition for active sites. Furthermore, the immobilized thin film mechanical robustness ensured that binding sites were accessible throughout the adsorption process. Overall, our findings indicate that, even at moderate surface charge, biosorption systems exhibit high metal affinity when supported by structurally accessible functional groups, favorable solution chemistry, and multi-modal sorption pathways.

#### 3.4.5 Scanning electron microscopy analysis

**Fig. 9:**
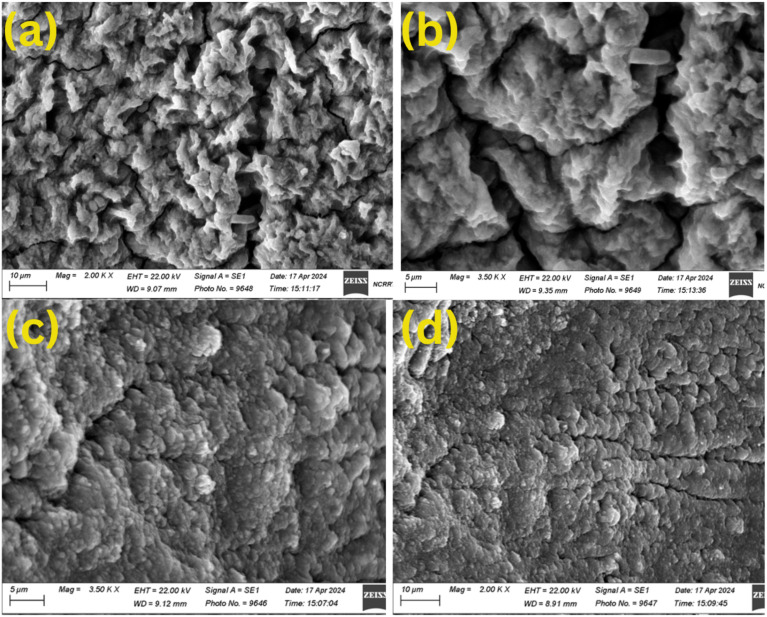
(a and b) SEM micrographs of *Chlorella sorokiniana* thin films before biosorption demonstrated that algal thin films exhibited an extremely varied surface, with high roughness, many protrusions, porous structures and irregular microcracks. These features lead to greater surface area and active sites, which explain why heavy metal adsorption is more effective. The image (a) was acquired at 2.00 KX magnification with a 10 µm scale bar, and a working distance of 9.07 mm. The image (b) was acquired at 3.50 KX magnification with a 5 µm scale bar, and a working distance of 9.35 mm. (c and d) SEM micrographs of *Chlorella sorokiniana* thin films after biosorption, were conducted at 80 ppm Cd^2+^ aqueous solution, revealed a smoother, denser, and more compact algal film surface, indicating the accumulation of metal ions in the algal matrix. The image (c) was acquired at a 5 µm scale bar, 3.50 KX, 22.00 kV EHT, and a 9.12 mm working distance. The image (d) was acquired at a 10 µm scale bar, 2.00 KX, 22.00 kV EHT, and 8.91 mm working distance.

The SEM examination revealed essential information about the morphological characteristics of *Chlorella sorokiniana* thin films before and after cadmium biosorption (conducted at 80 ppm Cd^2+^ aqueous solution). *Chlorella sorokiniana* thin films before treatment had an extremely varied surface, with deep longitudinal pores, high roughness, porosity, and irregular microcracks, as shown in Fig. 9. These surface characteristics dramatically boosted the specific surface area and number of active binding sites, increasing the likelihood of cadmium ion contact and entrapment. Surface roughness promotes microscale turbulence at the solid–liquid interface, resulting in more cadmium ion collisions and improved adsorption *via* diffusion and electrostatic interaction. Branching and linked pores facilitate capillary driven transport of Cd^2+^ into deeper film layers, creating a multidimensional diffusion pathway and boosting access to interior active regions. Microcracks and Cavities: These functioned as micro reservoirs, lengthening the residence time of cadmium ions at adsorption sites and increasing local concentration gradients, resulting in more surface contacts. Following cadmium biosorption (conducted at 80 ppm Cd^2+^ aqueous solution). SEM images revealed a smoother, denser, and more compact film surface, the observed microstructural alterations, such as densely aggregated spherical particles and decreased surface porosity, pointing to the accumulation of metal ions in the biomass matrix, as shown in Fig. 9. The morphological alterations indicate that Cd^2+^ substituted native cations, causing metal induced crosslinking with carboxyl, hydroxyl, and phosphate groups on algal cell walls. Cadmium was detected on the post treatment surfaces by SEM-EDX elemental mapping, validating the metal absorption efficiency of immobilized *C. sorokiniana* films. These findings suggest a biosorption process including physical trapping, chemical complexation, and ion exchange, resulting in a structurally reinforced biosorbent with high removal capability.

### 3.5 Biosorption kinetics and isotherm modeling

#### 3.5.1 Biosorption kinetics and statistical evaluation

Kinetic modeling was used to investigate the biosorption of Cd(ii) on immobilized *Chlorella sorokiniana* thin film. The analysis of the biosorption mechanism involved the application of three kinetic models: the pseudo-first-order kinetics, pseudo-second-order kinetics and intraparticle diffusion models through the fitting of experimental data. The data for kinetic parameters acquired at an initial concentration of 80 ppm at 80 ppm Cd^2+^ to challenge the biosorbent under high load conditions and to evaluate its maximum removal capacity, was presented in Table 5.

**Table 5:** Kinetic parameters of Cd^2+^ biosorption fitted to three different kinetic models^a^

Kinetic model	Parameter			
Pseudo-first order	*k*_1_ (h^−1^) = 0.0013	*q*_e_ (exp.) = 1.139	*q*_e_ (calc.) = 0.593	*R*_2_ = 0.0001
Pseudo-second order	*k*_2_ (g (mmol^−1^ h^−1^)) = 0.212	*q*_e_ (exp.) = 1.139	*q*_e_ (calc.) = 1.28	*R*_2_ = 0.984
Intraparticle diffusion	*k*_i_ (mmol (g^−1^ h 0.5)) = 0.14			*R*_2_ = 0.980

a *k*_1_: rate constant of the pseudo-first-order model; *k*_2_: rate constant of the pseudo-second-order model; *q*_e_: equilibrium adsorption capacity (mmol g^−1^); *R*^2^: correlation coefficient.

**Fig. 10:**
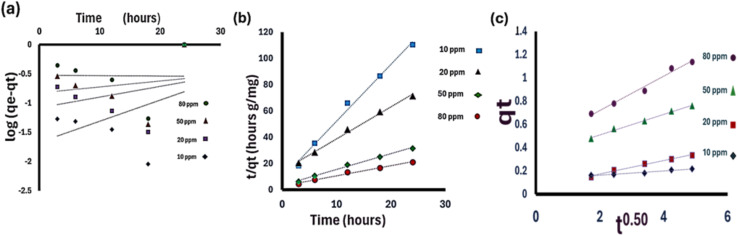
(a) Pseudo-first-order kinetic plots for Cd(ii) biosorption onto algal thin films under controlled conditions: initial concentrations (*C*_i_) = 10, 20, 50, and 80 ppm; pH = 7; temperature = 25 °C; biosorbent dose = 2.3 g L^−1^; contact times = 3, 6, 12, 18, and 24 hours. (b) Pseudo-second order plot for biosorption of Cd ions on to algal thin films under controlled conditions: initial concentrations (*C*_i_) = 10, 20, 50, and 80 ppm; pH = 7; temperature = 25 °C; biosorbent dose = 2.3 g L^−1^; contact times = 3, 6, 12, 18, and 24 hours. (c) Intraparticle diffusion model for biosorption of Cd ions on to algal thin films under controlled conditions: initial concentrations (*C*_i_) = 10, 20, 50, and 80 ppm; pH = 7; temperature = 25 °C; biosorbent dose = 2.3 g L^−1^; contact times = 3, 6, 12, 18, and 24 hours.

The pseudo-first-order model has a low correlation (*R*^2^ = 0.0001), resulting in a significant difference between calculated (*q*_e,calc._ = 0.593 mg g^−1^) and experimental uptake (*q*_e,exp._ = 1.139 mg g^−1^), Fig. 10. The regression analysis indicated a non significant slope (*p* = 0.8369) and too broad confidence ranges (−0.191 to 0.213), indicating that this model does not accurately represent the kinetics of Cd(ii) biosorption under the experimental conditions. The pseudo-second-order model accurately predicted experimental data, with a high coefficient of determination (*R*^2^ = 0.984) and low standard error (±0.801). The model produced a calculated equilibrium uptake (*q*_e,calc._ = 1.28 mg g^−1^) that was nearly identical to the experimental finding (*q*_e,exp._ = 1.139 mg g^−1^), demonstrating model consistency, as shown Fig. 10. The statistical analysis revealed a significant slope (*p* = 0.0067) with a narrow 95% confidence range (0.464–0.978) and an intercept *p*-value at the significance level (*p* = 0.058), implying that chemisorption is the dominant rate limiting mechanism in the biosorption process. This finding is corroborated by FTIR studies that show distinct interactions between cadmium ions and functional groups such as –OH, –COOH, and –NH. SEM-EDX and XRD imaging demonstrated that cadmium had been effectively deposited on the algal thin film surface. Overall, these data support the idea that the biosorption process involves valence forces or ion exchange, which is consistent with the pseudo-second-order model assumptions. The intraparticle diffusion model is strongly correlated with experimental data (*R*^2^ = 0.980), with a corrected *R*^2^ value of 0.957 and a low standard error of ±0.035, Fig. 10. The model's slope coefficient was statistically significant (*p* = 0.0144) with a narrow 95% confidence range (0.074–0.237), indicating that intraparticle diffusion plays a substantial role in the overall biosorption process. The intercept (*C* = 0.392) was also statistically significant (*p* = 0.0328), demonstrating that, the biosorption process encompasses many phases, including surface adsorption and internal diffusion. The results of all kinetic models suggest that Cd(ii) biosorption onto immobilized algal films is primarily regulated by chemisorption (as depicted by the pseudo-second-order model), with intraparticle diffusion playing a role in a multistage process. The statistical analysis verifies these findings, emphasizing the importance of understanding both surface and interior diffusion mechanisms in biosorption.

#### 3.5.2 Adsorption isotherm modeling and statistical evaluation

Four isotherm models were used: Langmuir, Freundlich, Sips, and Dubinin–Radushkevich, to study the equilibrium behavior of cadmium biosorption on immobilized *Chlorella sorokiniana* thin film. The data for isotherm parameters was presented in Table 6.

**Table 6:** Adsorption isotherm constants and parameters for Cd^2+^ biosorption^a^

Adsorption isotherm	Parameters		
Langmuir	*q*_m_ (mg g^−1^) = 1.38	*b* (L mg^−1^) = 0.13	*R*_2_ = 0.80
			*R*_L_ = 0.085
Freundlich	*k*_f_ (mg g^−1^) = 0.37	1/*n* = 0.75	*R*_2_ = 0.970
Dubinin–Radushkevich	*q*_m_ (mg g^−1^) = 1.02	*B* = 4 × 10^−6^	*R*_2_ = 0.90
			*E* = 354
Sips	*k*_s_ = 0.20	1/*n* = 0.53	*R*_2_ = 0.70

a *q*_m_ *:* maximum adsorption capacity (mg g^−1^); *b:* Langmuir constant related to binding energy (L mg^−1^); *R*^L^: separation factor; *K*_f_ and *n*: Freundlich constants; *β:* Dubinin–Radushkevich constant (mol^2^ kJ^−2^); *E*: mean adsorption energy (kJ mol^−1^); *K*_s_ and *n:* Sips model constants; *R*^2^*:* correlation coefficient.

**Fig. 11:**
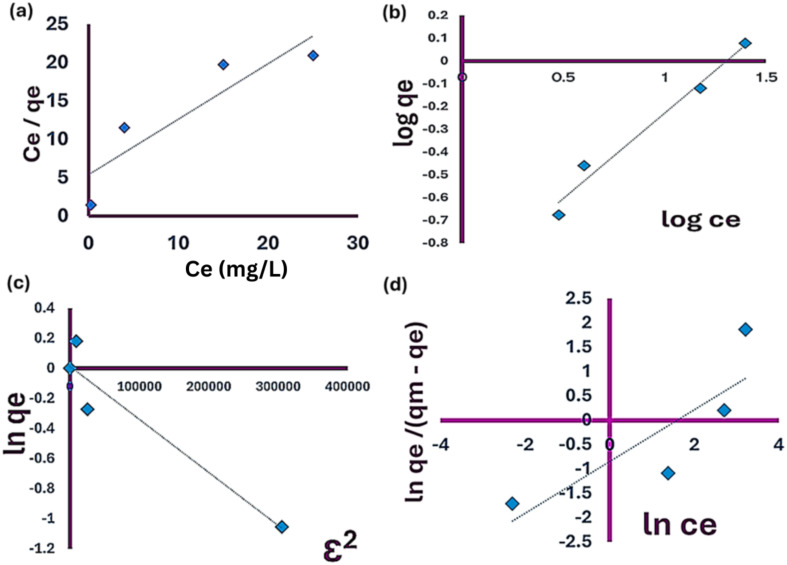
(a) Langmuir isotherm plot for biosorption of Cd ions on to algal thin films under controlled conditions: initial concentrations (*C*_i_) = 10–80 ppm; pH = 7; temperature = 25 °C; biosorbent dose = 2.3 g L^−1^; contact times = 3, 6, 12, 18, and 24 hours. (b) Freundlich isotherm plot for biosorption of Cd ions on to algal thin films under controlled conditions: initial concentrations (*C*_i_) = 10–80 ppm; pH = 7; temperature = 25 °C; biosorbent dose = 2.3 g L^−1^; contact times = 3, 6, 12, 18, and 24 hours. (c) Dubinin–Radushkevich isotherm plot for biosorption of Cd ions on to algal thin films under controlled conditions: initial concentrations (*C*_i_) = 10–80 ppm; pH = 7; temperature = 25 °C; biosorbent dose = 2.3 g L^−1^; contact times = 3, 6, 12, 18, and 24 hours. (d) Sips isotherm plot for biosorption of Cd ions on to algal thin films under controlled conditions: initial concentrations (*C*_i_) = 10–80 ppm; pH = 7; temperature = 25 °C; biosorbent dose = 2.3 g L^−1^; contact times = 3, 6, 12, 18, and 24 hours.

The Freundlich isotherm accurately described the experimental data, with the greatest correlation coefficient (*R*^2^ = 0.970), as shown in Fig. 11. This provides a viable multilayer adsorption approach for various surfaces, confirmed by the multilayer structure visible in SEM images. The statistical regression analysis verified this, with a low standard error for the intercept (±0.04), a narrow 95% confidence interval (0.80–1.82), and a statistically significant *p*-value (*p* = 0.0197). The slope has a standard error of ±1.51 with a 95% confidence interval of −0.35 to 3.36, indicating that the model is robust in predicting adsorption intensity (1/*n* = 0.75). The Langmuir model accurately predicted *q*_m_ = 1.38 mg g^−1^ and *b* = 0.13 L mg^−1^, with a *R*^2^ value of 0.80, as shown in Fig. 11. However, statistical analysis found wide confidence ranges for the slope (−1.84 to 2.75), intercept (−28.24 to 49.70), and non-significant *p*-values. These results indicate that the Langmuir model has limited credibility under current conditions, confirming the premise that cadmium biosorption does not occur in monolayers on a homogenous surface. The Dubinin–Radushkevich model has a *R*^2^ of 0.90, a theoretical maximum capacity (*q*_m_) of 1.02 mg g^−1^, *β* = 4 × 10^−6^ mol^2^ kJ^−2^, Fig. 11. The mean free energy (*E*) value estimated from the model was 354 kJ mol^−1^, which based on theoretical thresholds, indicated that the biosorption process is driven by particle diffusion and strong chemical interactions,.^53,207^ The slope had a large standard error (±0.26), a wide confidence interval (−2.97–3.50), and a non-significant *p*-value (*p* = 0.492). Even though the Dubinin–Radushkevich (D–R) model showed a non-significant *p*-value, it was included for internal comparison with other isotherm models and to be consistent with previous microalgal biosorption research, where the D–R model has frequently shown good fit and interpretive value.^208–212^ Including it here enables comparison with prior studies and indicates how the current system may behave differently in terms of sorption energetics. Also, the Dubinin–Radushkevich (D–R) isotherm model emphasizes the energy aspect of the adsorption process and is commonly used to differentiate between physical and chemical adsorption.^213^ The Sips model shows a poor correlation (*R*^2^ = 0.70), Fig. 11, and large 95% confidence intervals for intercept (−19.42 to 12.83) and slope (−4.82 to 7.79). Non-significant *p*-values indicate insufficient dependability in characterizing the experimental data. The obtained constants (*K*_s_ = 0.20, 1/*n* = 0.53) show heterogeneous adsorption, however with less statistical support than the Freundlich model.

### 3.6 Mechanistic perspectives on cadmium biosorption

**Fig. 12:**
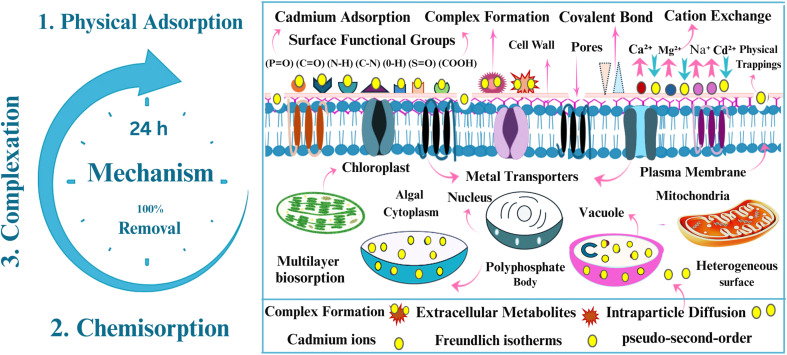
Mechanism of cadmium biosorption by *Chlorella sorokiniana* thin films is a multi-step process that includes early physical adsorption, intraparticle diffusion, cation exchange and strong chemisorption *via* functional group interactions, all without the formation of crystalline precipitates. (This illustration proposed based on this current study).

To gain insight into the underlying mechanisms of cadmium removal, the biosorption process was analyzed in terms of interactions between functional groups and Cd(ii) ions, supported by characterization data and modeling results. This finding of cadmium (Cd^2+^) biosorption by *Chlorella sorokiniana HMY-C* thin films represent a significant advancement in metal recovery technology. This study uses different analysis such as kinetic modeling, equilibrium isotherms, Fourier-transform infrared spectroscopy (FTIR), scanning electron microscopy-energy dispersive X-ray spectroscopy (SEM-EDX), X-ray diffraction (XRD), and zeta potential measurements to explore the complexities of the biosorption process. In the initial phase, rapid physical adsorption predominated, as demonstrated by a considerable increase in removal efficiency throughout the first three hours. This phase was differentiated by the algal thin film enormous surface area, roughness, and abundance of active binding sites, which allowed cadmium ions to collide and combine *via* electrostatic interactions. The accessibility of surface regions dominated the first physical adsorption process. As the contact time increased, the biosorption process became slower and more diffusion controlled. The SEM study revealed microcracks and branching holes in the algal films, indicating the emergence of multidimensional diffusion channels. Subsequently, the primary mechanism shifted to chemical binding. Equilibrium data fitting indicated that the Freundlich isotherm, which depicts a heterogeneous surface with multilayer adsorption, was the best model for cadmium biosorption. A kinetic examination confirmed these results, with a pseudo-second-order model indicating that chemisorption, aided by strong chemical interactions, took predominance in the late phases of biosorption. FTIR spectroscopy shows that cadmium biosorption involves interactions between Cd^2+^ ions and functional groups on algal thin film surfaces. Significant spectrum alterations and the creation of new bands revealed the presence of stable coordination complexes containing hydroxyl, amine, carbonyl, phosphate, and sulfonyl groups. The absence of ester carbonyl bands and the presence of thiocyanate and phosphodiester signals indicate complexation and multi-coordination bonding between Cd^2+^ ions and the algal thin films surface. XRD examination demonstrated that cadmium was kept inside the structure of the algae without the production of crystalline phases, highlighting the biosorption process's effectiveness in preventing the formation of precipitated cadmium phases. Overall, the results show that cadmium biosorption on *Chlorella sorokiniana* thin films involves multi-step process, including physical adsorption, intraparticle diffusion, cation exchange with Ca^2+^, and significant chemisorption through surface functional groups (*e.g.*, –COOH, –OH), Fig. 12. This synergistic, multi-mechanistic technique increases cadmium removal effectiveness, confirming algal thin films viability as a long-term potential for complex heavy metal remediation applications. Finally, the proposed mechanism is therefore strongly supported by the combined evidence obtained from kinetic, isotherm, spectroscopic, microscopic, and electrokinetic analyses.

## 4 Environmental implications and future perspectives

Based on the obtained results, the potential environmental applications and sustainability of the selected microalgal strain for heavy metal remediation are discussed, along with recommendations for future research.

### 4.1 Potential for multi-metal removal

In this study, based on FTIR spectroscopy, the SEM study and the isothermal study, the heterogeneity of the algal thin film surface, which is composed of various functional groups such as carboxyl, hydroxyl, phosphate, and amine groups, provides an excellent platform for binding a wide range of heavy metal ions. The present system's performance with Cd^2+^ demonstrates potential for large applications. Future research should look at competing adsorption characteristics and compare the selectivity and efficiency of *C. sorokiniana* thin films to different metal ions. Understanding these interactions will allow for the development of biosorbents that can resist extremely effluent conditions.

### 4.2 Regeneration and reuse of immobilized thin films

Based on the biosorption experiments conducted in this study, cadmium binding is mostly mediated by chemical interactions rather than precipitation, which allows for fast desorption and several reuses of the biosorbent. Immobilized *C. sorokiniana* thin films may be regenerated with simple desorbing agents, retaining their high biosorption capacity across several cycles. More study is required to assess the long term mechanical integrity and adsorption performance after several regenerations. This contributes to estimating the operating life of films in continuous treatment systems.

### 4.3 Post-use management and environmental safety

Considering the structural and functional properties observed in the present study, although the *Chlorella sorokiniana HMY-C* thin film is naturally biodegradable, post-use biosorbents contaminated with cadmium must be carefully controlled to avoid subsequent environmental contamination. Chemical desorption using mild acidic or chelating chemicals is the preferred method of eliminating bonded cadmium since the biosorbent may be reused. Following metal removal, the leftover biomass, which is still rich in energy-dense organic material, may be converted into biofuels under controlled conditions. This dual approach enables the safe disposal of hazardous waste while also conforming to circular bioeconomy principles by converting depleted biosorbents into renewable energy resources. To properly explore ecological compromises and scalability, future research should include life cycle assessment (LCA) and investigate the integration of biosorption bioenergy systems into industrial wastewater treatment facilities.

### 4.4 Broader implications for wastewater treatment

This research makes a substantial contribution to the development of sustainable, bio-based industrial wastewater treatment systems. It provides a strong scientific foundation for the use of immobilized algal films by combining kinetic, mechanistic, and surface level research. The system's great efficiency, reusability, and flexibility demonstrate its potential as a practical solution to real world environmental problems.

### 4.5 Scalability and industrial relevance

While the system has shown effective at eliminating cadmium in controlled laboratory settings, commercial use requires validation under dynamic flow and real effluent circumstances. A pilot-scale test with actual industrial effluent containing complicated metal combinations will be required. Furthermore, incorporating these thin films into current modular wastewater treatment frameworks might be a cost-effective and straightforward solution for businesses coping with heavy metal contamination. Furthermore, we propose incorporating this technology into modular treatment units driven by solar energy, which might boost its viability in scattered industrial settings, particularly in rural or resource-constrained locations with limited traditional infrastructure.

### 4.6 Limitations and durability challenges

Despite their promise, immobilized thin films may have limitations owing to biofouling, mechanical deterioration, and structural weaknesses. To address these issues, techniques such as surface modification with antimicrobial nanoparticles and alginate matrix strengthening with crosslinking agents are suggested to improve the films' mechanical and biological durability. Future research should focus on developing biosorption systems that can remove multiple metals at once, incorporating immobilized thin films into existing modular wastewater treatment plants, and improving the mechanical robustness and antifouling properties of biosorbent films to ensure long-term operation in dynamic, real-world environments.

### 4.7 Economic and environmental aspects of algal thin film biosorption

Based on the superior Cd removal efficiency established by the designed Chlorella sorokiniana thin-film system, this approach offers significant environmental and financial advantages for sustainable treatment of wastewater. The production cost of *Chlorella* sp*.* In dry wild type is ($ 2.3 to 2.5 kg^−1^); however, prices can be reduced by (30–40%) by supplementing nutrients with wastewater effluents. In previous study, the growth of microalgae on synthetic media for potential wastewater treatment applications, highlighting the economic and ecological advantages. The utilizing residential wastewater to alter macronutrient levels in the growth environment allowed for the production of biomass with a high protein content (45–57% dry weight).^214^*Chlorella sorokiniana* biomass included 53.25% proteins, 27.95% lipids, 14.25% carbohydrates, and 2.66% pigments, indicating its benefits as a feedstock to produce a variety of bioproducts.^215^ Microalgae biotechnology as a crucial aspect in achieving sustainable development goals Additionally, treated wastewater has been shown to meet FAO and WHO requirements for agricultural reuse, providing additional economic and environmental advantages.^216^ Moreover, the thin-film system's treatment cost (USD 0.80–1.11 per m^3^) is comparable to traditional and emerging technologies like activated carbon (USD 5–200 m^−3^,^98^ as cited in 99), chemical precipitation (USD 4 m^−3^,^77^ and nanotechnology) USD 6.35 m^−3^,^101^ highlighting its economic viability and potential for scalable application. These findings demonstrate the financial and ecological benefits of employing *Chlorella sorokiniana* based on thin films. In addition to conserving finance, this study promotes the principles of the circular bioeconomy. Future studies should focus on standardized techno-economic modeling, long term field scale validation, and regulatory integration for wastewater reuse in the industrial and agricultural sectors.

## 5 Conclusion

This study was designed to develop and evaluate a sustainable, highly effective biosorption system for removing cadmium from both synthetic and real industrial effluent using *Chlorella sorokiniana* thin films. All research objectives were achieved, confirming the technology's robustness and potential for scalable wastewater treatment. Under optimal operating conditions, the algal thin film removed 100% of cadmium at low concentrations and maintained high performance up to 80 ppm, exceeding traditional immobilized beads and suspended biosorbents. Kinetic and isotherm modeling revealed a multilayered and heterogeneous biosorption process that corresponded to the pseudo-second-order and Freundlich models. Mechanistic insights confirmed that cadmium uptake occurs in a sequence of physical adsorption, intraparticle diffusion, cation exchange, and strong chemisorption *via* functional group interactions, with additional structural trapping confirmed by FTIR, SEM-EDX, XRD, and zeta potential analyses all without the formation of crystalline precipitates. The biosorption efficacy of *C. sorokiniana* thin film was verified under actual industrial wastewater conditions (0.4, 2.4 ppm Cd^2+^), achieving full removal despite the presence of competing ions. This illustrates the system's remarkable selectivity and adaptability, which is unusual for laboratory-prepared synthetic systems. Demonstrating full-scale efficiency under real-world wastewater circumstances is a significant advance toward industrial implementation since it provides strong evidence of practical viability and environmental significance. Furthermore, immobilizing algal cells within the alginate matrix increased biosorption stability while simultaneously ensuring biosafety. Although changes in microbial community structure were not particularly studied, the immobilized *C. sorokiniana HMYA-C* thin film system was purposefully intended to limit algal leakage into the surrounding environment. This containment reduces ecological disturbance and guarantees that biosorption occurs without disrupting the natural microbial balance of the treated water. From a sustainability perspective, the suggested thin-film system combines high efficiency, low production costs, and environmental friendliness, making it a suitable platform for large-scale applications. With a focus on long-term performance, regeneration cycles, and sensitivity to several contaminants. Looking forward, shifting from batch to continuous-flow configurations is critical for assessing operational stability and industrial scalability. Additionally, integrating hybrid functionalized biopolymers or nanocomposite matrices may improve film durability, adsorption selectivity, and long-term stability. The use of gene-edited microalgae with high metal binding affinities could be a next-generation approach to precision-engineered biosorbents. Additionally, integrating life-cycle assessment and techno-economic studies is crucial for evaluating this system against standard treatment technologies and assuring its environmental and economic feasibility. Subsequently, this study proposes a comprehensive biosorption technique that not only achieves high cadmium removal efficiency but also respects to circular bioeconomy principles. The closed-loop approach, in which treated biomass is reused for biofuel or biopolymer production, encourages clean water availability, resource recovery, renewable energy generation, and carbon footprint reduction. Overall, these findings highlight *Chlorella sorokiniana* thin films as a next-generation, environmentally friendly, and economically sustainable biosorbent for real-world wastewater treatment. This work lays a strong platform for enhanced biosorption technologies that combine scientific innovation, practical scalability, and environmental responsibility, thereby accelerating the worldwide transition to a sustainable and circular bioeconomy. This study bridges the gap between laboratory-scale biosorption research and real-world industrial wastewater treatment by developing a scalable, safe, and sustainable algal-based system that challenges the practical limits of bioremediation. Paving way for a cleaner, greener, and more sustainable future.

## Author contributions

The authors confirm contribution to the paper as follows: Heba M. Youssef was responsible for conceptualization, methodology, experimental work, investigation, data curation, writing – original draft preparation, advanced scientific illustration, writing – review & editing, and final manuscript assembly. Fatma Mohamed provided conceptualization, writing – original draft, continuous guidance throughout the study, writing – review & editing, revising the original draft, validation, supervision, reviewing the results and approved the final version of the manuscript. Mohamed S. Abd Elhameed and Khaled N. M. Elsayed provided conceptualization, supervision, writing – review & editing, validation, reviewing the results and approved the final version of the manuscript. All authors have read and agreed to the published version of the manuscript.

## Conflicts of interest

The authors affirm that they possess no recognized competing financial interests or personal affiliations that may have seemingly impacted on the research presented in this paper.

## Data Availability

All data supporting the findings of this study are included within the main article. No additional datasets were generated or analyzed during the current study.
